# Systematic Evaluation of the Safety Threshold for Allograft Macrovesicular Steatosis in Cadaveric Liver Transplantation

**DOI:** 10.3389/fphys.2019.00429

**Published:** 2019-04-25

**Authors:** Zhengtao Liu, Junjun Jia, Huaijun Ning, Shuping Que, Lin Zhou, Shusen Zheng

**Affiliations:** ^1^Division of Hepatobiliary and Pancreatic Surgery, Department of Surgery, First Affiliated Hospital, School of Medicine, Zhejiang University, Hangzhou, China; ^2^Key Laboratory of Combined Multi-Organ Transplantation, Ministry of Public Health, First Affiliated Hospital, School of Medicine, Zhejiang University, Hangzhou, China; ^3^Collaborative Innovation Center for Diagnosis and Treatment of Infectious Diseases, Hangzhou, China; ^4^Department of Pediatrics, Women and Children's Hospital of Guangxi, Nanning, China; ^5^Science for Life Laboratory, KTH - Royal Institute of Technology, Stockholm, Sweden

**Keywords:** donor, macrovesicular steatosis, mortality, outcomes, liver transplantation

## Abstract

**Background:** Currently, 30% macrovesicular steatosis (MaS) content is usually assigned empirically as the boundary between “use” and “refuse” a donor liver for liver transplantation (LT); however, this cut-off is questionable due to the lack of systemic evidence of the efficiency relative to prognosis prediction. Clinicians have tried to identify the threshold for optimized utilization of marginal steatotic allografts, but controversy exists among different studies.

**Aim:** Our study aimed to systematically determine an acceptable donor MaS content cut-off without incurring extra risk in liver transplantation, using meta-analysis.

**Methods:** The relevant literature reporting the relationship between MaS content and post-transplant mortality/morbidity was searched and retrieved in Pubmed, Embase, and ISI Web of Science.

**Results:** Nine studies were enrolled into the final analysis. A categorical comparison revealed that patients who received allografts with moderate steatosis (MaS content >30%) had significantly higher risks of graft failure/dysfunction, but not of mortality. Dose-response analysis showed that donor MaS content affected the graft failure/dysfunction in a non-linear relationship. Risks associated with MaS content in terms of poorer outcomes were independent of other risk covariates for liver transplantation. A non-significant increase in risk of inferior post-transplant outcomes was observed in patients who received allografts with a MaS content <35%. The risks of post-transplant graft failure and dysfunction increased with severe donor MaS content infiltration, without a consistent relationship.

**Conclusions:** The threshold of allograft MaS content can be safely extended to 35% without additional risk burden on post-transplant inferior outcomes. Clarification on “the effects of stratification” for MaS content can provide theoretical evidence for further optimal utilization of marginal steatotic allografts in liver transplantation.

## Introduction

Liver transplantation (LT) is still the most effective strategy for treatment of end-stage liver disease, hepatobiliary carcinoma, and acute/chronic liver failure. Currently, organ shortage is prominent in view of the contradiction between the limited donor pool and the ongoing increasing demand for liver allografts from patients registered on waiting lists (Lucidi et al., 2015). A steatotic allograft is one of the most commonly used marginal donors in clinical practice (McCormack et al., 2011), but affects the prognosis in >30% of patients who undergo liver transplantation (McCormack et al., 2011). Given the global epidemic of non-alcoholic fatty liver disease (Younossi et al., 2018) and the increase in organs from older donors (Halazun et al., 2018), the impact of steatotic grafts on transplantation cases is an inevitable issue.

Fatty infiltration amplifies the susceptibility to primary non-function (PNF) and early allograft dysfunction (EAD) (McCormack et al., 2011; Deschenes, 2013); however, adverse events associated with implants have been presented inconsistently as a result of variance in pathologic type and steatotic severity (Attia et al., 2008). Currently, organs with mild macrovesicular steatosis (MaS) content (<30%) are routinely used in many centers without additional risk, while the risks associated with grafts with moderate (30–60%) or severe (>60%) MaS content are increased, but controversial, with discrepancies across different studies (Busuttil and Tanaka, 2003; Nocito et al., 2006). Some researchers have speculated the existence of a “threshold value” for MaS content in connection with a prominent impact on post-transplant outcomes, but the exact cut-off value is unknown (Imber et al., 2002).

Dozens of papers have been published over recent decades, evaluating the risk of MaS allografts on post-operational morbidity and mortality (Urena et al., 1998; Crowley et al., 2000; Verran et al., 2003; Burra et al., 2009; Li et al., 2009; Noujaim et al., 2009; Doyle et al., 2010; Deroose et al., 2011; da Teng et al., 2012; de Graaf et al., 2012; Chavin et al., 2013), but inconsistencies have been observed among studies regarding many aspects, including primary disease, steatosis classification, and outcome indicators. A dose-response meta-analysis provides the possibility of data integration, quantitative risk assessment, and cut-off evaluation (Orsini et al., 2011). In the current study, key issues were systematically elucidated, as follows: (1) trends and quantitative risk assessment of allograft MaS content as a continuous covariate in post-transplant outcomes as a function of time; (2) Identification of a “cut-off” value for MaS content triggering a significant increase in morbidity and mortality risk; and (3) potential confounders affecting the relationship between MaS content and post-transplant outcomes.

## Methods

### Search Strategy

This study was conducted strictly according to the guidelines of Preferred Reporting Items for Systematic Reviews and Meta-analyses (PRISMA) (Moher et al., 2009). A systematic literature search was performed in Pubmed, Embase, and ISI Web of Science using the medical terminologies described in Table S1, with the language restriction limited to English (updated until 20 January 2019). Details of the search strategy in each database are presented in Table S1.

### Inclusion Criteria

Given the study purpose, we attempted to conduct a broad search that included literature involving continuous risks of all post-operational outcomes in different time periods associated with different degrees of pre-operational allograft MaS severity. Full-length articles that met the following inclusion criteria were included as eligible literature: (1) study was based on adult patients (age >18-years) who received cadaveric liver transplantation; (2) graft MaS content was determined by histological examination before implanting into recipients; (3) patients were categorized into three or more groups by donor MAS severity; (4) MaS-specific patient mortality and graft failure were reported or could be evaluated by calculation; (5) patients were followed for >90-days; and (6) sample size was >50 participants for each individual study.

### Data Extraction

Available information for all eligible studies was extracted independently by two investigators (ZL and SQ) according to a unified standardized reporting form. Potential inter-investigator discrepancies were checked and resolved by a third experienced author (HN).

Information collected for the extraction form included the following items, if provided in the original literature: (1) general information (author, country of origin, publication date, duration of follow-up, and definition of post-operative complications/ symptoms); (2) allograft factors (steatotic severity and methods used for histological examination); (3) etiology for liver transplantation and post-operative cause of death; (4) donor/recipient factors [age, gender distribution, body mass index (BMI), and model of end-stage liver disease (MELD) score]; (5) surgical factors (operational data and surgical approaches); and (6) MaS-specific outcomes [post-operative laboratory examination, length of hospitalization/intensive care unit (ICU) stay, occurrence rates of EAD/PNF, and patient mortality/organ failure].

Data involved in graphs of enrolled literature were extracted by GetData Graph Digitizer software (v 2.26; downloaded from http://getdata-graph-digitizer.com/index.php. For studies providing only a MaS content range, the median value was defined as the mid-point across the upper and lower limits. For open-ended data, the median value was 20% higher than the lower limit or 20% lower than the upper limit.

### Quality Assessment

Methodologic quality was assessed by two reviewers (ZL and SQ) independently for each study, based on items from the Newcastle-Ottawa Scale (NOS) for non-randomized cohort study (NOS checklist) (Wells et al., 2016) (http://www.ohri.ca/programs/clinical_epidemiology/oxford.asp). Specifically, enrolled studies were assessed by NOS under the following three conditions: (1) patient selection; (2) group comparability; and (3) definition of the exposure or outcome of interest quantified by the star system. A study awarded six or more stars were considered high quality based on the NOS system.

### Data Synthesis and Statistical Analysis

Odds ratios (ORs) were chosen to estimate the risk of MaS content on patient mortality, organ failure, and related post-operational complications. If the data was not provided in the enrolled literature, the OR and corresponding 95% confidence interval (CI) were evaluated using an online calculator (https://www.medcalc.org/calc/oddsratio.php) based on the case and total number of patients in different MaS content categories.

For a categorical comparison, the combined OR was evaluated in patients who received organs with different MaS content [(high and middle) vs. low]. Pooled standardized mean differences (SMDs) were chosen to assess the quantitative differences in groups classified by graft MaS content (high/middle vs. low). The calculation was performed by Metan (Higgins et al., 2003). The dose-response impact of allograft MaS severity on post-transplant outcomes was evaluated using a two-stage random-effects dose-response model, which was developed by Orsini et al. (2011) Specifically, dose-response ORs were modeled by restricted cubic spline in a fixed-effect model with fixed knots at 5, 35, 65, and 95% of the MaS distribution. First, the restricted cubic spline model was constructed using generalized least-squares regression with consideration of the log OR and relevant variance in each individual study (Orsini et al., 2006). Then, the separate ORs were combined by a multivariate random-effects model (Jackson et al., 2010). Pooled data were plotted to show the risk tendency. Evidence of non-linearity was examined by null hypothesis on regression coefficients in pooled cubic splines (equal to zero). A *P* < 0.05 was assumed to be significant for a non-linear relationship. Compared to groups using allografts without MaS, the extent of risk was assessed at fixed knots of the MaS content based on best curves. For non-linear cubic splines, the safety threshold was defined as the donor MaS content with a lower 95% CI of the OR on post-transplant outcomes at 1.

### Subgroup Analysis, Meta-Regression, Sensitivity Analysis, and Publication Bias Analysis

Subgroup analysis was performed to separately evaluate the effects of potential confounders on the risk associated with MaS severity on patient and graft survival rates. Sensitivity analysis was conducted to assess the influence of a single study on the overall effects of pooled results. Potential publication bias was estimated by Egger's test (Egger et al., 1997). The influence of intermediate confounders on the association between MaS content and post-transplant outcomes was determined by meta-regression (Higgins and Thompson, 2004)^27^. Statistical heterogeneity was evaluated based on chi-squared Q and *I*^2^ tests. *I*^2^ values of 25, 50, and 75% were defined as low, moderate, and high heterogeneity, respectively (Higgins et al., 2003). Calculations were performed using Stata software (release 14; StataCorp, College Station, TX, USA) and a two-sided *P*-value (*P* < 0.05) was considered to be statistically significant.

## Results

### Literature Selection and Study Flow-Diagram

**Figure 1:**
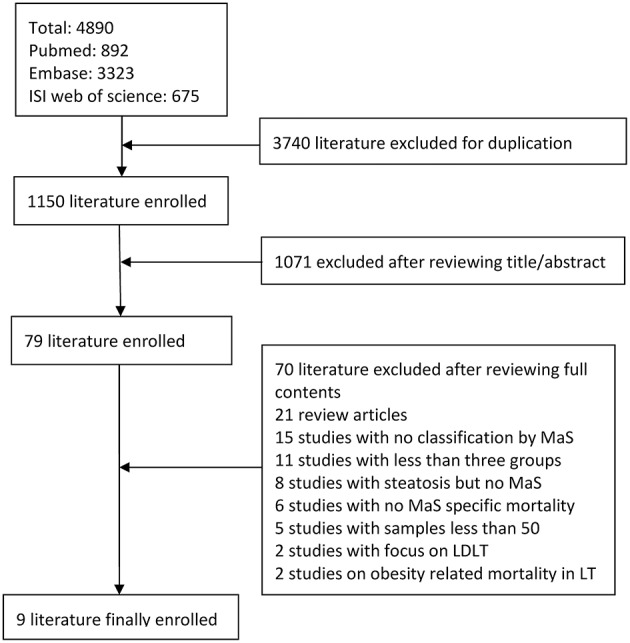
Study flow diagram for selection of qualified studies. MaS, macrovesicular steatosis; LDLT, living donor liver transplantation.

Figure 1 shows a flow-diagram, illustrating the procedure for selection of the literature. Nine studies involving the risk of stratified MaS content on post-transplant outcomes were enrolled into our analysis. High consistency across investigators were observed in the literature retrieval (kappa index = 0.81).

### Quality Assessment

The NOS scale for a non-randomized cohort study was adopted for quality assessment. The majority of studies had high-quality performance based on results from the NOS scale (score ≥ 6). Two studies (Verran et al., 2003; Nikeghbalian et al., 2007) had inferior quality, which was mainly due to defects in comparability and ascertainment of post-transplant outcomes (Table S2).

### Description of Enrolled Studies

The characteristics of enrolled studies are shown in Table 1. Nine studies (Verran et al., 2003; Briceño et al., 2005; Nikeghbalian et al., 2007; Burra et al., 2009; Li et al., 2009; Noujaim et al., 2009; Doyle et al., 2010; Deroose et al., 2011; de Graaf et al., 2012) with 1,976 patients were included for a meta-analysis. The patients received cadaveric liver transplantations between 1993 and 2008. The patient age range was 45–55-years. The average duration of follow-up was 21–86 months. Five studies provided gender distribution data; male gender was dominant (range, 55.0–75.8%). Five studies reported the indications for liver transplantation and showed no hierarchical variation in underlying diseases in groups receiving grafts with different MaS content. All enrolled data originated from a single-center study. Liver biopsies were performed to evaluate steatosis post-reperfusion in most studies (six of nine); liver biopsies were obtained before reperfusion in three additional studies (Table 1). MaS content was quantitatively evaluated and determined based on the percentage of large droplets of fat on hematoxylin and eosin (H&E)-stained slides under microscopic observation.

**Table 1:** Characteristics of enrolled studies.

						**Recipients**					**Donors**			**Surgery**		
**References, country**	**Surgical Period**	**Follow-up (mon)**	**MaS** **(%)**	**Number**	**Time for Liver Biopsy**	**Etiology**	**Age** **(yr)**	**Male (%)**	**BMI** **(kg/m^**2**^)**	**MELD-score**	**Age**	**BMI** **(kg/m^**2**^)**	**Male** **(%)**	**Surgical type**	**CIT (min)**	**WIT (min)**
(de Graaf et al., 2012), Australia	2001–2007	na	0	71	Post-reperfusion	ALC = 10 Hepatic failure = 5 CHC = 17 CHB = 4 HCC = 5 PBC = 6 PSC = 4 Others = 15	50 ± 12	na	na	19 ± 7	42 ± 16	24 ± 4	52.1	OLT/cadaveric liver grafts	466 ± 140	na
			1–30	59		ALC = 6 Hepatic failure = 5 CHC = 18 CHB = 2 HCC = 10 PBC = 4 PSC = 3 Others = 7	53 ± 9			19 ± 8.6	48 ± 15	28 ± 6	72.8		510 ± 138	
			31–60	7		ALC = 1 CHC = 3 HCC = 1 PSC = 1 Others = 1	52 ± 5			14 ± 4.7	37 ± 11	26 ± 4	71.4		463 ± 169	
			>60	4		HCC = 1 Others = 3	47 ± 12			15 ± 6.6	55 ± 6	33 ± 9	75		582 ± 34	
(Deroose et al., 2011), Netherland	2000–2004	86 (0–127)	0	92	Before/Post reperfusion	^a^VH = 51 ALC = 12 CC = 17 Billiary disease = 28 Others = 42	46 ± 1^a^	56.0^a^	24.9 ± 0.4^a^	18 ± 1^a^	na	na	na	OLT/cadaveric liver graft	504 for DCD	na
			1–30	39											432 for non-DCD	na
			31–60	19												
			>60	15		^b^VH = 5 ALC = 0 CC = 3 Billiary disease = 4 Others = 3	48 ± 3^b^	73.3^b^	24.0 ± 0.5^b^	18 ± 3^b^						
(Doyle et al., 2010), USA	2002–2008	25 (4–86)	<5	222	Before/Post-reperfusion	VH = 101 CC = 28 ALC = 27 NAFLD = 11 Billiary disease = 24 Others = 31	55 (18–75)	67.5	27.7 ± 5.4	20 (6–50)	45 (10–80)	31 (17–53)	na	OLT/cadaveric liver graft	342 (84–876)	na
			5–35	66		VH = 101 CC = 28 ALC = 27 Billiary disease = 4 Others = 11	55 (35–76)	80.3	29.1 ± 4.6	20 (6–50)	50 (19–75)	32.7 (21.5–41.9)			366 (96–750)	
			35–60	22		VH = 13 CC = 1 ALC = 1 NAFLD = 1 Billiary disease = 1 Others = 5	49 (22–66)	77.2	29.0 ± 5.6	22 (11–36)	39 (18–69)	31.4 (17.4–53.3)			330 (138–594)	
(Noujaim et al., 2009), Brazil	2002–2008	21 ± 19.5	<5	34	Post-reperfusion	na	51 ± 11	na	na	22 ± 8	45.4 ± 13.6	25 ± 2.5	na	Cadaveric liver graft	614 ± 161	na
			5–30	12			52 ± 14			19 ± 10	42.7 ± 12.7	26 ± 3.3			623 ± 189	
			30–60	6			47 ± 12			21 ± 12	43 ± 12.6	25 ± 3.3			595 ± 167	
			>60	5			54 ± 8			21 ± 7	41 ± 13	25 ± 3.5			566 ± 142	
(Li et al., 2009), China	2003–2008	30.5 ± 5.2	1–20	29	Not referred	VH = 13 Billiary disease = 11 HCC = 5 Others = 3	46 (26–66)	65.5	23.7 ± 4.1	20 ± 10	35.4 (23–56)	24.5 ± 6.4	82.8	OLT/cadaveric liver graft	373 ± 76	na
		33.7 ± 6.4	21–40	23		VH = 13 Billiary disease = 6 HCC = 4 Others = 3	43 (33–65)	73.9	22.1 ± 3.7	19 ± 8	38.1 (22–52)	26.1 ± 6.1	78.3		342 ± 66	
		32.4 ± 5.7	41–60	18		VH = 8 Billiary disease = 7 HCC = 3 Others = 3	45 (28–62)	61.1	24.7 ± 3.5	20 ± 10	35.4 (25–52)	29.7 ± 4.3	88.8		355 ± 80	
(Burra et al., 2009), Italy	1999–2001	36	0^c^	59	Not referred	^d^CHC = 56 CHB = 18 Billiary disease = 14 ALD = 28	47.4 ± 10.7	70.6^d^	na	17 ± 5.2	26.5 ± 12.3	na	63.8^d^	na	442 ± 98	59 ± 10
			1–33	46			48.8 ± 9.3			17.5 ± 6.2	39.4 ± 14.8				470 ± 141	57 ± 9
			>33	11			51.4 ± 6.74			18.7 ± 5.0	37.3 ± 12.3				454 ± 104	56 ± 13.4
(Nikeghbalian et al., 2007), Iran	1993–2006	25.7 ± 26.6	0–10	90	Not referred	na	na	na	na	na	na	na	na	OLT/cadaveric liver graft	na	na
		26.8 ± 22.5	10–30	50												
		22.8 ± 19.3	30–60	34												
(Briceño et al., 2005), Spain	na	na	0	255	Before/Post-reperfusion	ALD = 125 CHC = 111 Cholestatic liver disease = 43 CHB = 34 CC = 29 Other = 158	52 (18–72)	75.8	na	17.25	na	na	na	OLT/cadaveric liver graft	187 ± 48	48 ± 16
			1–30	160						17.4					183 ± 42	43 ± 14
			30–60	67						14.5					159 ± 62	42 ± 19
			>60	18						14.4					131 ± 50	42 ± 14
(Verran et al., 2003), Australia		na	0	323	Post-reperfusion	na	na	na	na	na	34	na	na	OLT/cadaveric liver graft	na	na
			1–30	72							43 (S1/S2/S3)					
			>31	48												

*Data was presented as mean ± SD or median (range)*.

a *represented patients received grafts with steatosis ≤ 60%*.

b *represented patients received grafts with steatosis>60%*.

c *represented patients including HCV and non-HCV infectors*.

d *represented the features including all enrolled recipients or donors*.

*ALC, alcoholic liver cirrhosis; ALD, alcoholic liver disease; BMI, body mass index; CC, cryptogenic Cirrhosis; CHB, chronic hepatitis B; CHC, chronic hepatitis C; CIT, cold ischemia time; DCD, donation after cardiac death; HCC, hepatocellular cell carcinoma; MaS, macrovesicular steatosis; MELD, model for end-stage liver disease; na, not available; NAFLD, non-alcoholic fatty liver disease; OLT, orthotopic liver transplantation; PBC, primary biliary cirrhosis; PSC, primary sclerosing cholangitis; VH, viral hepatitis; WIT, warm ischemia time*.

The MaS-specific post-transplant indicators reported in each individual study are summarized in Table S3. Six studies reported both patient and organ survival in the enrolled cohort. Another three studies reported only the patient or graft survival data. Of note, three studies excluded allografts with severe MaS content (>60%) as an absolute contraindication for liver transplantation. Of the patients, 1,146 (58%), 556 (28%), and 214 (11%) received organs with low (MaS content <10%), mild (10% < MaS content <30%), and moderate (30% < MaS content <60%) steatosis, respectively. The patients in six studies who received organs with severe steatosis (MaS content >60%) were combined (60 patients; <3% of all cases). Data on the development of PNF and EAD were available in five and six studies, respectively.

Varied causes of patient death and graft loss were summarized in five studies (Table S4). The most common cause for patient death was post-operative infection/sepsis (47%), recurrence of primary disease (21%), and bleeding (16%) 3-years after liver transplantation. Organ failure was usually attributed to patient death (46%), hepatic artery thrombosis (14%), and graft rejection (19%). When categorized by MaS severity, potent risk factors that had been raised in prior predictive models on post-transplant prognosis [donor/recipient age, BMI, MELD score, and cold ischemic time (CIT)] were not comparable. Graft MaS severity was positively correlated with donor age and BMI (*P* < 0.05, Table S5). Grafts with a higher MaS content tended to select for recipients with lower MELD scores and shorter cold ischemic time; however, significant heterogeneity was presented across individual studies for the above-mentioned positive results (*I*^2^> 50%, *P* < 0.05; Table S5). Publication bias was assessed based on the cold ischemic time between groups with higher and lower MaS content (*P* for Egger's test = 0.02). MaS content was associated with the peak level of liver enzymes [alanine aminotransferase/aspartate aminotransferase (ALT/AST)] and length of hospitalization (ward/ICU stay) after liver transplantation in four studies (Table S3). EAD and PNF were selected as major complications for observation. Diagnostic criteria were defined in six studies (Table S6). The definition of these complications varied greatly across individual studies.

### Impact of MaS Severity on Post-operational Mortality and Complications

The overall 90-day, 1, 2, 3, and 5-years post-transplant mortality rates were 12, 13, 15, 16, and 25% in the lower MaS content group, respectively. In the middle MaS content group, the corresponding mortality rates were 15, 16, 17, 21, and 25%, respectively. The post-transplant mortality rates increased to 20, 22, 22, 27, and 30% in the higher MaS content group, respectively.

The 90-day, 1, 2, 3, and 5-years post-operative graft loss rates were 13, 16, 16, 23, and 29% in the lower MaS content group, respectively. The graft loss rates were similar in the middle MaS content group (14, 17, 17, 23, and 30%, respectively), but increased in the higher MaS content group (24, 30, 29, 36, and 40%, respectively). EAD occurrence increased from 11 to 49% following transplantation with severe organ steatosis. An increasing trend was also observed regarding the incidence of PNF following transplantation with organs with elevated MaS content, but the total number of cases was much lower in the enrolled cohorts (<2%).

A categorical comparison was performed to evaluate the MaS-stratified risk of post-operative outcomes (Table 2). Data from the lower MaS content group was assigned as the reference for comparison. The number of available participants for comparison varied from 470 patients (in two studies) to 1,121 patients (in five studies; Table 2). Severe MaS significantly affected the 90-day patient mortality [pooled OR: 1.55 (1.03–2.35), *P* < 0.05], but beyond this result, donor allograft steatosis did not affect patient mortality in any post-transplant periods (*P* > 0.05). With respect to graft survival, no significant difference was found for the middle MaS content group compared to controls at different time points after liver transplantation (all *P* > 0.05); however, graft failure in the higher MaS control group increased disproportionately in the first 3-years after liver transplantation (Table 2). Severe steatosis (MaS > 30%) persistently increased the graft failure rate by approximately 2-fold in the following 3-years after liver transplantation with low-to-moderate heterogeneity (*I*^2^ ranged, 0–33.9%; *P* > 0.05). The pooled ORs for 90-day, 1, 2, and 3-years graft mortality rates in the severe MaS content group were 2.16, 2.47, 2.11, and 2.00, respectively. With respect to post-transplant complications, EAD and PNF were significantly associated with severe steatosis (MaS content >30%). Compared to the control group, severe MaS caused a 4-fold higher increase in EAD and PNF. Pooled ORs for the impact of MaS content on EAD are presented with evident inter-group heterogeneity across individual studies (*I*^2^ > 50%; *P* < 0.05, Table 2).

**Table 2:** Categorical comparison on post-transplant mortality and post-operational complications classified by macrosteatosis degree.

**Item**	**Comparison (MaS degree)**	**Number of studies**	**Number of patients**	**Pooled OR**	***I*^**2**^ (%)**	***p*-value (Heterogeneity chi-squared)**	***p*-value (Egger's test)**
**PATIENT MORTALITY**							
90-day	High vs. low	7	1,288	1.55 (1.03–2.35)	0	0.654	0.09
	Middle vs. low			1.23 (0.87–1.95)	25.2	0.237	0.04
1-year	High vs. low	6	892	1.63 (0.98–2.71)	0	0.761	0.14
	Middle vs. low			1.25 (0.80–1.95)	0	0.525	0.12
2-year	High vs. low	4	659	1.78 (0.98–3.26)	0	0.648	0.59
	Middle vs. low			1.03 (0.61–1.74)	0	0.616	0.70
3-year	High vs. low	3	589	1.78 (0.96–3.30)	0	0.397	0.60
	Middle vs. low			1.21 (0.73–1.98)	0	0.798	0.14
5-year	High vs. low	2	470	1.32 (0.69–2.53)	0	0.987	na
	Middle vs. low			0.98 (0.58-1.64)	0	0.415	na
**ALLOGRAFT FAILURE**							
90-day	High vs. low	5	1,472	2.16 (1.44–3.24)	33.9	0.195	0.96
	Middle vs. low			1.04 (0.71–1.51)	31.4	0.212	0.13
1-year	High vs. low	6	1,178	2.47 (1.61–3.80)	0	0.745	0.58
	Middle vs. low			1.21 (0.82–1.80)	0	0.440	0.18
2-year	High vs. low	5	1,141	2.11 (1.38–3.22)	0	0.411	0.87
	Middle vs. low			1.05 (0.75–1.53)	0	0.430	0.88
3-year	High vs. low	4	1,038	2.00 (1.28–3.13)	22.2	0.278	0.39
	Middle vs. low			1.13 (0.79–1.62)	0	0.765	0.75
5-year	High vs. low	3	1,055	1.51 (0.99–2.31)	0	0.453	0.40
	Middle vs. low			1.11 (0.79–1.54)	0	0.531	0.79
**POST-TRANSPLANT COMPLICATION**							
EAD	High vs. low	4	816	4.02 (2.14–7.53)	73.2	0.011	0.42
	Middle vs. low			1.28 (0.70–2.34)	73.2	0.011	0.06
PNF	High vs. low	7	1,762	4.26 (1.54–11.8)	0	0.560	0.36
	Middle vs. low			1.57 (0.52–4.76)	0	0.891	0.53

*Data in extremely higher MaS group (MaS > 60%) was combined for comparison. Egger's test can't be performed when lower than three studies was enrolled for analysis (na). EAD, early allograft dysfunction; MaS, macrovesicular Steatosis; OR, odds ratio; PNF, primary non-function*.

Egger's test revealed that publication bias was not significant in nearly all comparisons, except for slight asymmetry for the 90-day patient mortality rate in a comparison between the middle and lower MaS content groups (*P* = 0.04; Table 2).

The differences in post-transplant liver enzymes and length of hospitalization cannot be combined due to an absence of standard deviation (SD) values in the original studies. All studies revealed prominent increases in liver enzyme peaks (ALT/AST) in the higher MaS content group (*P* < 0.05; Figure S1). For post-operative hospitalization, two studies speculated that higher graft steatosis (MaS content >30%) might prolong the length of the ward and ICU stay, but a negative relationship was presented in another three studies (Figure S1).

### “Safety Threshold” of MaS Content in Liver Transplantation

**Figure 2:**
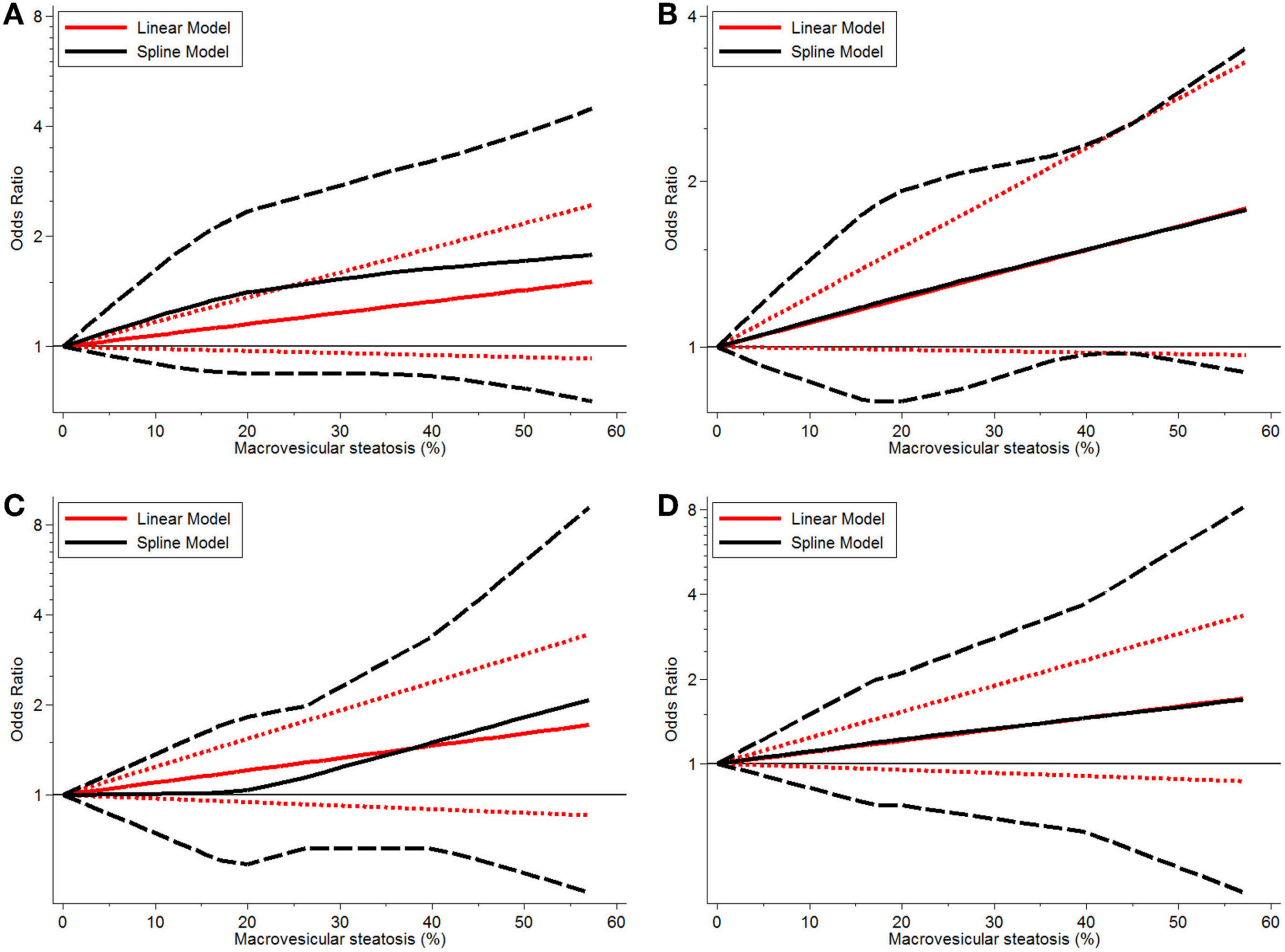
Dose-response relationship between donor MaS degree and the risk of patient mortality. **(A)** Dose-response relationship between donor MaS degree and the risk of 90-days patient mortality. **(B)** Dose-response relationship between donor MaS degree and the risk of 1-year patient mortality. **(C)** Dose-response relationship between donor MaS degree and the risk of 2-year patient mortality. **(D)** Dose-response relationship between donor MaS degree and the risk of 3-year patient mortality. The black solid and long-dashed curves represented instant ORs and their respective 95% CIs for patients' mortality compared to the subgroup using allografts without MaS based on the restricted cubic splines model. The red solid and short-dashed line represented the instant ORs and their respective 95% CIs for patients' mortality compared to the subgroup using allografts without MaS based on the generalized least squares model. MaS, macrovesicular steatosis; CI, confidence interval; OR, odds ratio.

**Figure 3:**
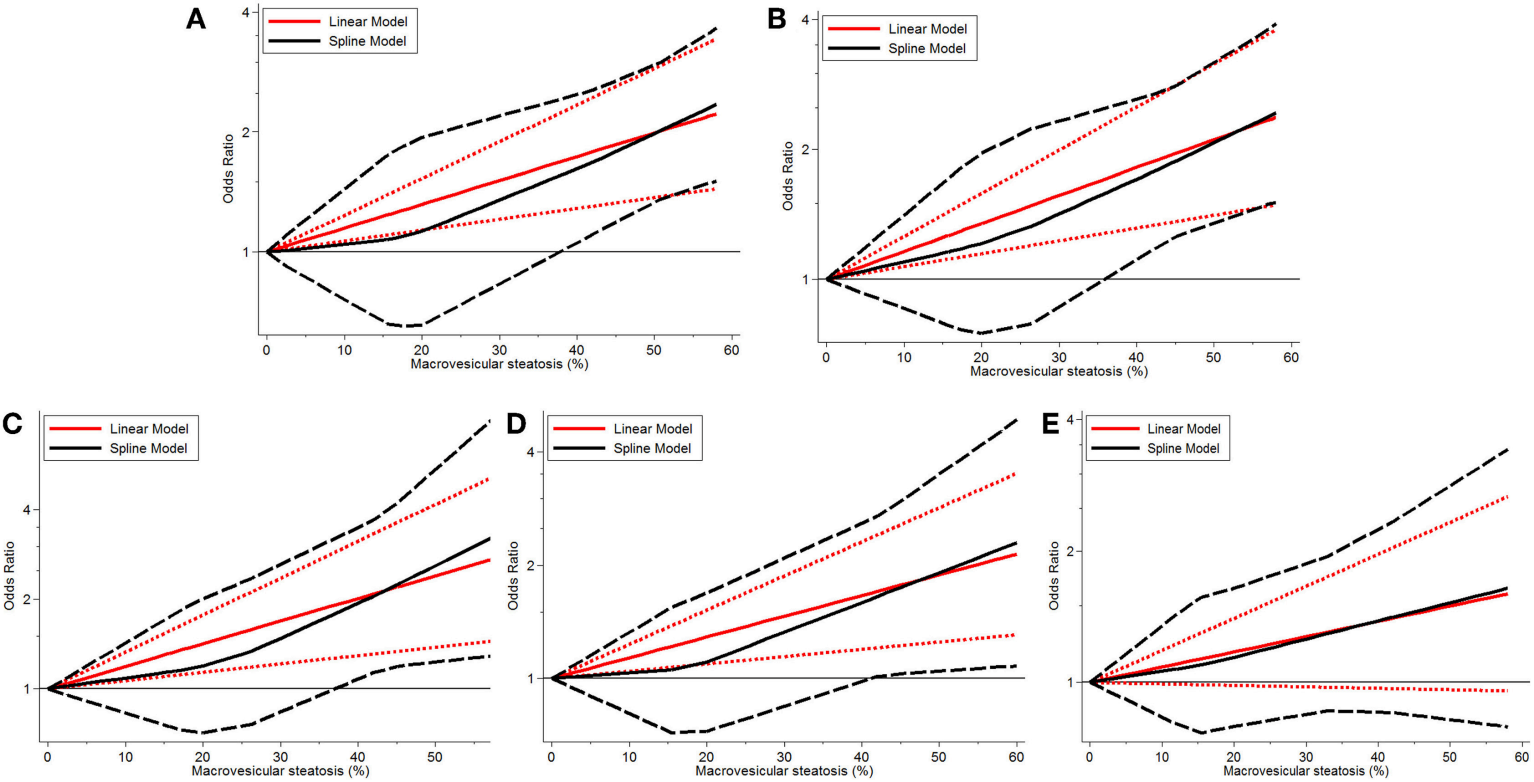
Dose-response relationship between donor MaS degree and the risk of allograft failure. **(A)** Dose-response relationship between donor MaS degree and the risk of 90-day allograft failure. **(B)** Dose-response relationship between donor MaS degree and the risk of 1-year allograft failure. **(C)** Dose-response relationship between donor MaS degree and the risk of 2-year allograft failure. **(D)** Dose-response relationship between donor MaS degree and the risk of 3-year allograft failure. **(E)** Dose-response relationship between donor MaS degree and the risk of 5-year allograft failure. The black solid and long-dashed curves represented instant ORs and their respective 95% CIs for allograft failure compared to the subgroup using allografts without MaS based on the restricted cubic splines model. The red solid and short-dashed line represented the instant ORs and their respective 95% CIs for allograft failure compared to the subgroup using allografts without MaS based on the generalized least squares model. MaS, macrovesicular steatosis; CI, confidence interval; OR, odds ratio.

Continuous dose-response risk of allograft MaS content on post-transplant outcomes was evaluated by best-fit spline (Figures 2, 3). The number of patients and studies for each assessment is listed in Table 3.

**Table 3:** Dose-response risk for MaS severity on post-transplant outcomes.

**Item**	**Number of study**	**Number of participant**	***P* for non-linearity**	**Regression model/Pooled OR^c^ (95%CI)**	***P* for heterogeneity**	***P* for significance^a^**	**MaS safety threshold (%)^b^**
**Patient mortality**							
90-day	7	1,463	0.22	GLS	0.39	0.10	na
10 vs. 0%				1.07 (0.99–1.17)			
20 vs. 0%				1.15 (0.97–1.36)			
30 vs. 0%				1.23 (0.96–1.59)			
40 vs. 0%				1.33 (0.95–1.86)			
50 vs. 0%				1.42 (0.94–2.17)			
60 vs. 0%				1.53 (0.92–2.54)			
1-year	6	892	0.18	GLS	0.48	0.08	na
10 vs. 0%				1.11 (0.99–1.24)			
20 vs. 0%				1.22 (0.99–1.51)			
30 vs. 0%				1.38 (0.98–1.87)			
40 vs. 0%				1.50 (0.98–2.30)			
50 vs. 0%				1.66 (0.97–2.84)			
60 vs. 0%				1.84 (0.96–3.49)			
2-year	4	659	0.59	GLS	0.09	0.13	na
10 vs. 0%				1.10 (0.97–1.24)			
20 vs. 0%				1.21 (0.95–1.54)			
30 vs. 0%				1.33 (0.92–1.91)			
40 vs. 0%				1.46 (0.90–2.38)			
50 vs. 0%				1.60 (0.87–2.95)			
60 vs. 0%				1.76 (0.85–3.67)			
3-year	3	589	0.99	GLS	0.11	0.12	na
10 vs. 0%				1.10 (0.97–1.24)			
20 vs. 0%				1.21 (0.95–1.53)			
30 vs. 0%				1.33 (0.93–1.89)			
40 vs. 0%				1.46 (0.90–2.34)			
50 vs. 0%				1.60 (0.88–2.90)			
60 vs. 0%				1.76 (0.86–3.59)			
**Organ failure**							
90-day	5	1,472	<0.01	RCS	0.07	n.a	38.0
10 vs. 0%				1.02 (0.70–1.55)			
20 vs. 0%				1.13 (0.67–1.89)			
30 vs. 0%				1.33 (0.81–2.14)			
40 vs. 0%				1.61 (1.05–2.46)			
50 vs. 0%				1.98 (1.33–2.98)			
60 vs. 0%				2.44 (1.54–3.85)			
1-year	6	1,178	<0.01	RCS	0.20	n.a	35.0
10 vs. 0%				1.08 (0.80–1.53)			
20 vs. 0%				1.22 (0.78–1.90)			
30 vs. 0%				1.42 (0.90–2.22)			
40 vs. 0%				1.70 (1.11–2.61)			
50 vs. 0%				2.07 (1.35–3.19)			
60 vs. 0%				2.53 (1.55–4.11)			
2-year	6	1,141	<0.01	RCS	0.29	n.a	36.0
10 vs. 0%				1.06 (0.76–1.62)			
20 vs. 0%				1.21 (0.74–1.93)			
30 vs. 0%				1.48 (0.88–2.35)			
40 vs. 0%				1.93 (1.08–3.37)			
50 vs. 0%				2.59 (1.25–5.47)			
60 vs. 0%				3.50 (1.30–9.16)			
3-year	4	1,042	<0.01	RCS	0.37	n.a	42.0
10 vs. 0%				1.01 (0.76–1.38)			
20 vs. 0%				1.11 (0.73–1.68)			
30 vs. 0%				1.31 (0.83–2.01)			
40 vs. 0%				1.58 (0.97–2.56)			
50 vs. 0%				1.92 (1.08–3.46)			
60 vs. 0%				2.29 (1.07–4.88)			
5-year	4	1,059	0.10	GLS	0.09	0.08	n.a
10 vs. 0%				1.08 (0.99–1.18)			
20 vs. 0%				1.17 (0.98–1.40)			
30 vs. 0%				1.27 (0.98–1.66)			
40 vs. 0%				1.38 (0.97–1.97)			
50 vs. 0%				1.50 (0.96–2.33)			
60 vs. 0%				1.61 (0.96–2.76)			
**Complications**							
EAD	4	816	<0.01	RCS	<0.01	n.a	38.6
10 vs. 0%				1.13 (0.45–3.75)			
20 vs. 0%				1.41 (0.33–5.29)			
30 vs. 0%				1.90 (0.57–6.12)			
40 vs. 0%				2.67 (1.09–6.86)			
50 vs. 0%				3.78 (1.80–8.11)			
60 vs. 0%				5.30 (2.64–10.5)			
PNF	7	1,762	<0.01	RCS	0.67	n.a	39.5
10 vs. 0%				1.07 (0.51–2.73)			
20 vs. 0%				1.26 (0.40–3.66)			
30 vs. 0%				1.73 (0.60–4.70)			
40 vs. 0%				2.65 (1.03–6.84)			
50 vs. 0%				4.20 (1.61–11.1)			
60 vs. 0%				6.53 (2.28–18.4)			

*Evaluation can't be performed for patients' 5-year mortality for less than three studies reported the relevant data*.

a *P-value for statistical significance was only evaluated in GLST model*.

b *Safety threshold was defined as the cut-off MaS value with lower limit of 95% CI at 1 in RCS model*.

c *Regression model was selected for each indicators (e.g., 90-day patient mortality), Pooled OR was listed as risk extent for difference on MaS severity in each specific indicator (e.g., 10% vs. 0% in 30-day patient mortality)*.

*GLS, generalized least-squares; MaS, macrovesicular steatosis; RCS, Restricted cubic splines*.

For MaS-specific patient mortality, a linear model was used for non-significance of the non-linearity test of regression coefficients in cubic splines (all *P* > 0.05). As shown in Figure 2, there was a trend of increasing patient mortality in patients who received allografts with severe MaS content. In response to a 10% increment of liver fatty infiltration, 7, 11, 10, and 10% higher risks were observed on 90-day, 1, 2, and 3-years patient mortality rates, but with statistical non-significance compared to controls (all *P* > 0.05). More details on pooled ORs and 95% CIs of different donor MaS contents on patient survival in distinguished time points are listed in Table 3. No heterogeneity was observed across individual studies (*P* > 0.05).

Donor MaS content affected the post-transplant organ failure in a non-linear pattern (*P* for non-linearity <0.05). As shown in Figure 2 and Table 3, a non-significant increment on organ mortality occurred in patients who received organs with a lower MaS content (<30%) as the widely acceptable safety threshold for liver transplantation. The pooled ORs were 1.33 (95% CI: 0.81–2.14), 1.42 (95% CI: 0.90–2.22), 1.48 (95% CI: 0.88–2.35), 1.31 (95% CI: 0.83–2.01), and 1.02 (95% CI: 0.67–1.51) for 90-day, 1, 2, 3, and 5-years allograft mortality, respectively. In addition, the safety threshold of MaS content can be extended to 38, 35, 36, and 42% according to the impact on 90-day, 1, 2, and 3-years organ survival, respectively (Figure 2). The stratified risk of allograft MaS severity on post-transplant organ mortality is presented in Table 3. Non-significant heterogeneity was observed in the relationship between MaS content and organ survival (*P* > 0.05).

**Figure 4:**
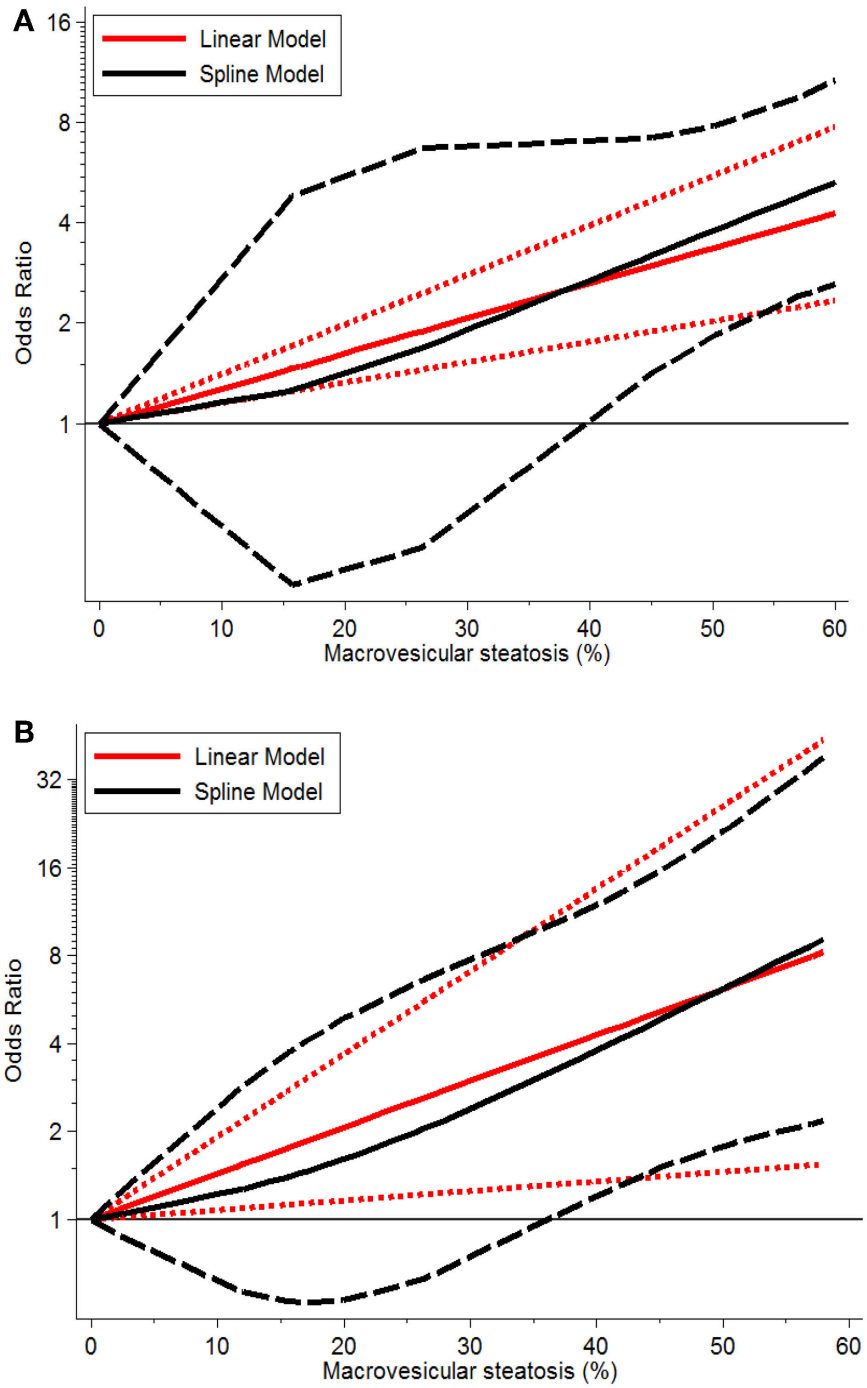
Dose-response relationship between donor MaS degree and the risk of post-operative complications. **(A)** Dose-response relationship between donor MaS degree and the risk of post-operative EAD occurrence; **(B)** Dose-response relationship between donor MaS degree and the risk of post-operative PNF occurrence. The black solid and long-dashed curves represented instant ORs and their respective 95% CIs for post-operative complications compared to the subgroup using allografts without MaS based on the restricted cubic splines model. The red solid and short-dashed line represented the instant ORs and their respective 95% CIs for post-operative complications compared to the subgroup using allografts without MaS based on the generalized least squares model. MaS, macrovesicular steatosis; CI, confidence interval; OR, odds ratio.

Severe MaS positively affected the development of EAD or PNF in a non-linear dose-response manner with prominent heterogeneity observed across individual studies (both *P* < 0.05, Table 3). An increase in post-transplant complications was observed in association with severe allograft MaS content. Compared to the group using negative steatotic grafts, 30% fatty infiltration caused a 1.90- and 1.73-fold higher occurrence of EAD and PNF, respectively (*P* > 0.05). The safety threshold for MaS severity on the incidence of EAD or PNF can be extended to 39 and 40%, respectively (Figure 4).

Except for the above-mentioned pooled risk, more details on the ORs of stratified allograft MaS severity on post-transplant patient/organ mortality in distinguished time-points are presented in Table 3.

### Comprehensive Analysis on Potential Confounders

As shown in Table 4, no significant inter-subgroup differences were demonstrated with respect to patient survival (*P* > 0.05). Of note, the results were still stable and no positive pooled risk of MaS content on patient mortality was observed in any subset.

**Table 4:** Subgroup analysis for risk assessment of macrovesicular steatosis on post-transplant mortality and complications.

	**Number of studies**	**Number of patients**	**OR^c^ (95%CI)**	***I*^**2**^ (%)**	***P*_1_^a^**	***P*_1_^b^**
**Patient mortality**						
Sample size						
< 150	3	243	2.44 (0.86–6.90)	0	0.73	
>150	3	649	1.43 (0.80–2.57)	0	0.54	0.38
Recipient age (years)						
<50	3	351	1.78 (0.78–4.09)	0	0.84	
≥50	2	367	2.50 (0.98–6.37)	0	0.66	0.60
Donor age (years)						
<40	2	186	1.89 (0.47–7.71)	0	0.56	
≥40	2	367	2.50 (0.98–6.37)	0	0.66	0.75
Recipient MELD score
<20	3	351	1.78 (0.78–4.09)	0	0.84	
≥20	2	367	2.50 (0.98–6.37)	0	0.66	0.60
Cold ischemic time (min)						
<400	2	380	2.33 (0.81–6.73)	0	0.72	
≥400	3	338	1.95 (0.90–4.19)	0	0.73	0.79
**Organ failure**						
Sample size						
<150	3	268	3.98 (1.48–10.7)	0	0.95	
>150	3	918	2.21 (1.37–3.56)	0	0.47	0.30
Recipient age (years)						
<50	2	235	1.47 (0.58–3.71)	0	0.46	
≥50	3	508	3.15 (1.46–6.77)	0	0.76	0.21
Donor age (years)						
<40	2	513	2.71 (1.47–4.97)	0	0.83	
≥40	3	508	3.15 (1.46–6.77)	0	0.76	0.76
Recipient MELD score						
<20	3	376	1.84 (0.83–4.07)	0	0.50	
≥20	2	367	3.06 (1.27–7.40)	0	0.47	0.40
Cold ischemic time (min)						
<400	2	380	2.57 (0.95–6.94)	0	0.79	
≥400	3	380	2.18 (1.05–4.54)	19.7	0.29	0.79
Time for liver biopsy						
Post-perfusion	3	641	2.97 (1.72–5.10)	0	0.76	
Before and post-perfusion	2	475	1.71 (0.82–3.57)	0	0.39	0.24
**Primary non-function**						
Sample size						
<200	4	492	4.68 (1.20–18.2)	0	0.50	
>200	3	1,253	3.43 (0.72–16.5)	7.6	0.34	0.77
Recipient age (years)						
<50	3	351	2.86 (0.63–13.0)	0	0.90	
≥50	3	951	12.3 (2.02–75.1)	6.1	0.35	0.23
Donor age (years)						
<40	3	629	2.21 (0.45–10.88)	0	0.85	
≥40	2	451	8.41 (0.95–74.7)	43.1	0.19	0.33
Recipient MELD score						
<19	4	922	6.36 (1.64–24.7)	10.4	0.34	
≥19	2	380	3.06 (0.33–28.5)	0	0.68	0.58
Cold ischemic time (min)						
<400	3	880	6.30 (1.01–39.4)	0	0.49	
≥400	3	442	4.61 (1.03–20.6)	14.7	0.31	0.80
Time for liver biopsy						
Post-perfusion	3	584	4.81 (0.79–29.2)	60.5	0.11	
Before/Post-perfusion	2	975	4.23 (0.96–18.7)	0	0.42	0.37

a *P_1_ value represented heterogeneity in subgroups*.

b *P_2_ value represented heterogeneity across subgroups*.

c *Pooled OR represented the risk with comparison performed on patient mortality, graft failure, or Primary non-function occurrence at 1-year in groups with higher (>30%) and lower (<10%) MaS degree. MELD, model for end-stage liver disease; OR, odds ratio*.

The pooled risk of graft survival was higher in studies with relatively older recipients (pooled OR: 3.15; 95% CI: 1.46–6.77) or patients with a higher MELD score (pooled OR: 3.06; 95% CI: 1.27–7.40), but did not reach statistical significance (*P* > 0.05, Table 4). Of note, subgroup analysis did not detect an interactive effect between prolonged cold ischemic time and the risk of MaS severity on graft loss in patients after liver transplantation. Subgroup analysis did not detect any factors with significant influence on PNF occurrence (all *P* > 0.05).

Considering the selection bias for recipient, donor, and surgical features categorized by MaS severity (Table S5), meta-regression was performed to evaluate the impact of inter-subgroup recipient, donor, or surgical difference on MaS-specific mortality/complications. As shown in Table S7, the inter-subgroup SMDs (for donor/recipient age, MELD score, and CIT did not affect the risk of patient mortality, allograft failure, and post-operational complications (all *P* > 0.05).

For sensitivity analysis on pooled risks of severe steatotic allografts, consistent trends were observed in most positive results by re-analysis after omitting each single study (Figures S2–S4). The pooled risk of MaS on PNF occurrence had a normal skewed distribution (Figure S4).

## Discussion

This is the first evidence-based study with a systematic and quantitative analysis of the risk associated with allograft macrovesicular steatosis as a risk covariate on post-operational clinical outcomes in patients who received orthotropic liver transplantation. Based on data combined in 1,976 cases of cadaveric liver transplantation from nine centers recorded from 2003 to 2011, we had major findings as follows: (1) The use of donor livers with mild steatosis (MaS content <30%) is safe without additional risk compared to non-steatotic grafts. (2) Moderate and severe donor liver steatosis (MaS content >30%) mainly affects the graft, but not patient survival in the first 3-years after liver transplantation. (3) Severe donor steatosis (MaS content >30%) also caused inferior prognosis by increasing the occurrence of EAD and PNF 4-fold in patients after liver transplantation. (4) Donor MaS affected the post-transplant allograft loss and complications in a non-linear, dose-dependent pattern. The threshold of donor MaS content can be safely extended to 35% for liver transplantation. (5) Allograft steatosis exerted its effect on post-transplant outcomes independent of other common risk covariates.

Followed with the rising prevalence of NAFLD in general population, steatosis has become a common and increasing phenomenon in candidate allografts for liver transplantation (Boteon et al., 2018; Moosburner et al., 2018). Allograft steatosis can be categorized into microvesicular, macrovesicular, and mixed forms on pathological perspective (Tannapfel et al., 2011). The microsteatosis (MiS) is usually considered as benign symptom without extra risk on post-transplant prognosis (Han et al., 2014a,b; Andert et al., 2017). Previous study found MiS didn't have interaction with MaS, either (Han et al., 2015). Severe MaS increased the post-transplant complications for its impact on ischemia/reperfusion injury and portal vein blood flow (Selzner et al., 2006). But the safety threshold was present with controversy across different studies (McCormack et al., 2011).

Disparities in MaS-specific graft failure and patient death have rarely been discussed in prior studies. Although trends are observed for both variables, we found that the pathologic MaS content of donor livers mainly exerted effects on post-operative graft failures, but not patient deaths. A more than 2-fold higher rate of organ failure occurred in the severe MaS content group 3-years after liver transplantation. With respect to patient survival, the MaS-specific risk was discrete and attenuated with increasing follow-up duration (Tables 2, 3). As shown in Table S4, patient death contributed to part of the etiology of graft failure (Lozanovski et al., 2018). We speculate that the risk of MaS-related graft failures might be mediated by non-fatal cases. All in all, our results indicated that the graft-failure-free survival might be more sensitive and accurate for post-transplant outcomes of steatotic donors.

On the background of global controversy between limited “organ source” and the increased “waiting list demand” (Pais et al., 2016), many centers have reached consensus that the allografts should be treated by distinction of categorical MaS severity. In general, donor livers with mild steatosis (MaS content < 30%) were acceptable for expansion of the donor pool without additional risk on post-transplant outcomes. Ongoing debate involves the impact of donor livers with moderate steatosis severity (MaS content ranging between 30 and 60%) with respect to wide discrepancies in terms of prognostic indicators after liver transplantation (Pais et al., 2016; Vodkin and Kuo, 2017; Vinaixa et al., 2018). While allografts with severe steatosis (MaS content >60%) were routinely discarded on the grounds of disproportionate increase in severe post-transplant complications, with the only exception being some special donor-recipient matched cases under additional restricted control on other risk indices like CIT or MELD scores (Chavin et al., 2013; Wong et al., 2016). In accordance with the previous consensus, our study revealed that allografts with mild MaS content (<30%) can be safely utilized without extra risk (Table 2), while less experience and unreliable results were aggregated for rare relevant data assessed in donors with severe steatosis (MaS content >60%) from enrolled studies [<3% of the total cases (data not shown)]. A key point in optimizing the use of steatotic livers involves the application of allografts with moderate MaS severity. Increased risk of organ loss and EAD/PNF occurrence was indicated in the group using grafts with moderate MaS contents (Table 2). Further dose-response analysis found stratified intra-group risks on post-transplant outcomes for moderate MaS content in a non-linear manner. The cut-off percentage of MaS content in the donor liver can be safely expanded to approximately 35% based on a predicted value fitted by the RCS model. Similarly, a very recent binary comparison of 611 patients from German clinical centers also confirmed the increased risk of 3-year graft failure with a threshold of 40% for MaS content (Lozanovski et al., 2018).

Primary graft dysfunction (PGD) is one of the major complications for patients after liver transplantation. Classified by disease severity, the post-transplant PGD can be divided into mild EAD and severe PNF (Deschenes, 2013; Chen and Xu, 2014; Neves et al., 2016). The existence of donor MaS was considered a major cause of PGD occurrence in a previous study (Kulik et al., 2017). No unified diagnostic criteria were defined across different centers (Table S6). The risk threshold was conventionally defined as 30% by experience in most cases without strong evidence grade (Davis and Florman, 2014; Neves et al., 2016). In our study, a 4-fold higher risk of EAD and PNF occurrence was observed in the group with severe steatotic allografts (MaS content >30%). Dose-response analysis revealed that the safety of the MaS cut-off could be extended to approximately 40%. A lower prevalence of PNF in all patients should be noted. The accurate PNF occurrence deviated from 1% in the lower MaS content group to 4% in the higher MaS content group.

Current concepts of donor quality appraisal emphasize that graft steatosis should be assessed as a continuous risk for liver transplantation rather than simply defined by “good” or “poor” quality (Durand et al., 2008). Therefore, dose-response risks of MaS severity on post-transplant outcomes at different time points were evaluated based on predicted values by best-fit curves (Table 3). In general, a trend of increasing post-transplant outcomes was observed to be associated with MaS development. Statistically significant risk increases in post-transplant graft failure or complications appeared to occur between 35 and 42% of MaS content, respectively; however, the grafts with a higher MaS content than the safety threshold should not be considered as an absolute contraindication for liver transplantation. Patient death might occur while on the waiting list (Kim et al., 2008) and the application of ECDs was proven to be an effective approach to reducing waiting list mortality by providing more sub-optimal organ donations for liver transplantation (Barshes et al., 2007). Use of allografts with severe MaS might further relieve the urgent organ shortage, with more opportunity of liver transplantation for recipients in the early stage of liver disease after registration, at the price of inferior post-transplant survival. Based on previous data, our study provided systemic data on evaluating the impact of steatotic donors on post-transplant mortality, which might help clinicians to make better decisions on the balance between the “risk” for MaS-related inferior survival and the “benefit” of saving more registrants from waiting list mortality.

Previous studies have shown additive effects of MaS on post-transplant outcomes by interaction with other risk factors. Briceño et al. (2005) found non-significant risk stratification on post-transplant mortality contributed by MaS severity in patients with lower MELD scores. Another case-control study reported similar effects of grafts with moderate MaS content on post-transplant prognosis compared to a non-steatotic group under strict limitation of CIT length (<8 h) (Westerkamp et al., 2015). Highly stringent selectivity on low-risk donors, recipients, and surgical options guaranteed the quality of inferior steatotic donors for liver transplantation (Wong et al., 2016). Based on pooled results, we found that the grafts with moderate and severe MaS content were usually allocated to patients with less severe disease under more stringent limitations on ischemia-reperfusion injury (Table S5); however, the impact of MaS content appeared stable on subgroup analysis classified by potential confounders (Table 4). No variation was observed on pooled risk of MaS-related post-transplant outcomes, even after adjustment for other risk factors, such as donor age, recipient MELD score, and intra-operative CIT (Table S7). Our data indicated independence on the risk of allograft MaS content on post-transplant outcomes, which cannot be compensated for by attenuation of other co-existing risks.

The strengths of our data are as follows: (1) Donor MaS content exerted an independent dose-dependent impact on post-transplant graft failure after adjustment for differences on other risk variables. (2) MaS content cut-off can be safely extended to 35% with a non-significant risk increase in post-operative mortality. Currently, approximately 20% of steatosis was observed with inter-regional variation in cadaveric organ donors (Koneru and Dikdan, 2002). In contrast, approximately 40% of donor livers with severe pathologic steatosis were discarded for liver transplantation, with steatosis being the primary cause, based on data from the European organ procurement organizations (Loinaz and Gonzalez, 2000). Allograft macrovesicular steatosis is considered an independent predictor for inferior outcomes and should be involved in risk assessment score systems for liver transplantation (Spitzer et al., 2010; Dutkowski et al., 2012). A defatting system, like machine perfusion, is utilized as an effective strategy to extend the pool of marginal steatotic donors (Boteon et al., 2018). Our data provided systematic evidence on risk index of donor MaS content in liver transplantation, which might be applied to facilitate the accuracy of further risk algorithms on outcome prediction, and to optimize the therapeutic destination for defatting devices.

The limitations of our study should be noted as follows. Firstly, all patients were enrolled from a single-center study. An individual study with fewer patients might limit the reliability of pooled results. Secondly, the severity of allograft steatosis was decided by H&E-stained biopsied samples in all studies. The H&E stain has lower accuracy in terms of prediction of steatotic severity for high inter-observer variation (Fiorini et al., 2004). Computerized analysis of Oil Red O (ORO)-stained samples were recommended to optimize the classification and minimize these random errors (Fiorini et al., 2004; Homeyer et al., 2017). Thirdly, potential bias might be caused by unavailable information on allograft biopsies in a portion of the donors. Fourthly, heterogeneity of the risk of post-transplant EAD occurrence cannot be assessed by subgroup analysis for less relevant data. The non-unified definition of post-transplant complications (Table S6) also contributed to inconsistency of pooled data, especially for EAD risk (Table 2). Pooled risks cannot be assessed on variables like length of ward/ICU stay for the absence of standard deviation. Fifthly, many factors, such as recipient age, MELD score, CIT length, and liver biopsy time, might interfere with the final results as potential confounders; however, subgroup analysis revealed that these covariates could not change the trend of results with a non-significant impact. Finally, selection bias was also attributed by an inter-subgroup difference in other major risk covariates (Table S5). The risk of MaS content on post-transplant outcomes might be mediated by aged and obese donors as other covariates for higher risk of inferior post-transplant outcomes (Bertuzzo et al., 2017); however, the results from meta-regression analysis revealed that the impact was negligible (Table S7).

In conclusion, allograft MaS content can be safely extended to 35% with acceptable post-transplant outcomes in cadaveric liver transplantation. Severe donor MaS content exerted its impact on graft failure and PGD, which was independent of other risks, including recipient MELD score or CIT length. A mechanistic study is warranted to further clarify the “threshold effects” of donor MaS content on post-transplant complications.

## Author Contributions

ZL and SZ conceived and designed the study. ZL, HN, and SQ extracted information and analyzed the data. ZL and JJ wrote the manuscript. HN, LZ, and SZ reviewed the manuscript. All authors approved the final manuscript for submission.

### Conflict of Interest Statement

The authors declare that the research was conducted in the absence of any commercial or financial relationships that could be construed as a potential conflict of interest.

## References

[B1] Andert A. Ulmer T. F. Schoning W. Kroy D. Hein M. Alizai P. H. (2017). Grade of donor liver microvesicular steatosis does not affect the postoperative outcome after liver transplantation. Hepatobiliary Pancreat. Dis. Int. 16, 617–623. 10.1016/S1499-3872(17)60064-X 29291781

[B2] Attia M. Silva M. A. Mirza D. F. (2008). The marginal liver donor–an update. Transplant. Int. 21, 713–724. 10.1111/j.1432-2277.2008.00696.x 18492121

[B3] Barshes N. Horwitz I. Franzini L. Vierling J. Goss J. (2007). Waitlist mortality decreases with increased use of extended criteria donor liver grafts at adult liver transplant centers. Am. J. Transplant. 7, 1265–1270. 10.1111/j.1600-6143.2007.01758.x 17359503

[B4] Bertuzzo V. R. Cescon M. Odaldi F. Di Laudo M. Cucchetti A. Ravaioli M.. (2017). Actual risk of using very aged donors for unselected liver transplant candidates. Ann. Surg. 265, 388–396. 10.1097/SLA.0000000000001681 28059967

[B5] Boteon Y. L. Boteon A. Attard J. Mergental H. Mirza D. F. Bhogal R. H.. (2018). *Ex situ* machine perfusion as a tool to recondition steatotic donor livers: troublesome features of fatty livers and the role of defatting therapies. A systematic review. Am. J. Transpl. 18, 2384–2399. 10.1111/ajt.14992 29947472

[B6] Briceño J. Padillo J. Rufián S. Solórzano G. Pera C. (2005). Assignment of steatotic livers by the Mayo model for end-stage liver disease. Transplant. Int. 18, 577–583. 10.1111/j.1432-2277.2005.00091.x 15819807

[B7] Burra P. Loreno M. Russo F. P. Germani G. Galligioni A. Senzolo M.. (2009). Donor livers with steatosis are safe to use in hepatitis C virus–positive recipients. Liver Transplant. 15, 619–628. 10.1002/lt.21761 19479805

[B8] Busuttil R. W. Tanaka K. (2003). The utility of marginal donors in liver transplantation. Liver Transplant. 9, 651–663. 10.1053/jlts.2003.50105 12827549

[B9] Chavin K. D. Taber D. J. Norcross M. Pilch N. A. Crego H. McGillicuddy J. W.. (2013). Safe use of highly steatotic livers by utilizing a donor/recipient clinical algorithm. Clin. Transplant. 27, 732–741. 10.1111/ctr.12211 23991646

[B10] Chen X.-B. Xu M.-Q. (2014). Primary graft dysfunction after liver transplantation. Hepatobiliary Pancreat. Dis. Int. 13, 125–137. 10.1016/S1499-3872(14)60023-0 24686540

[B11] Crowley H. Lewis W. D. Gordon F. Jenkins R. Khettry U. (2000). Steatosis in donor and transplant liver biopsies. Hum. Pathol. 31, 1209–1213. 10.1053/hupa.2000.18473 11070113

[B12] da Teng H. Zhu Z. J. Zheng H. Deng Y. L. Sun L. Y. Pan C.. (2012). Effect of steatosis donor liver transplantation on hepatocellular carcinoma recurrence: experience at a single institution. Hepatogastroenterology 59, 858–862. 10.5754/hge12007 22389257

[B13] Davis E. G. Florman S. S. (2014). Primary non-function, in Mount Sinai Expert Guides: Hepatology: Hepatology, eds Ahmad J. Friedman S. L. Dancygier H. (New York, NY: Wiley-Blackwell), 462–468.

[B14] de Graaf E. L. Kench J. Dilworth P. Shackel N. A. Strasser S. I. Joseph D.. (2012). Grade of deceased donor liver macrovesicular steatosis impacts graft and recipient outcomes more than the Donor Risk Index. J. Gastroenterol. Hepatol. 27, 540–546. 10.1111/j.1440-1746.2011.06844.x 21777274

[B15] Deroose J. P. Kazemier G. Zondervan P. IJzermans J. N. Metselaar H. J. Alwayn I. P. (2011). Hepatic steatosis is not always a contraindication for cadaveric liver transplantation. HPB 13, 417–425. 10.1111/j.1477-2574.2011.00310.x 21609375 PMC3103099

[B16] Deschenes M. (2013). Early allograft dysfunction: causes, recognition, and management. Liver Transplant. 19(Suppl. 2):S6–S8. 10.1002/lt.23746 24038766

[B17] Doyle M. M. Vachharajani N. Wellen J. R. Anderson C. D. Lowell J. A. Shenoy S.. (2010). Short-and long-term outcomes after steatotic liver transplantation. Arch. Surg. 145, 653–660. 10.1001/archsurg.2010.119 20644128

[B18] Durand F. Renz J. F. Alkofer B. Burra P. Clavien P. A. Porte R. J.. (2008). Report of the Paris consensus meeting on expanded criteria donors in liver transplantation. Liver Transplant. 14, 1694–1707. 10.1002/lt.21668 19025925

[B19] Dutkowski P. Schlegel A. Slankamenac K. Oberkofler C. E. Adam R. Burroughs A. K.. (2012). The use of fatty liver grafts in modern allocation systems: risk assessment by the balance of risk (BAR) score. Ann. Surg. 256, 861–869. 10.1097/SLA.0b013e318272dea2 23095632

[B20] Egger M. Smith G. D. Schneider M. Minder C. (1997). Bias in meta-analysis detected by a simple, graphical test. BMJ 315, 629–634. 9310563 10.1136/bmj.315.7109.629 PMC2127453

[B21] Fiorini R. N. Kirtz J. Periyasamy B. Evans Z. Haines J. K. Cheng G.. (2004). Development of an unbiased method for the estimation of liver steatosis. Clin. Transplant. 18, 700–706. 10.1111/j.1399-0012.2004.00282.x 15516247

[B22] Halazun K. J. Rana A. A. Fortune B. Quillin R. C. III. Verna E. C. Samstein B.. (2018). No country for old livers? Examining and optimizing the utilization of elderly liver grafts. Am. J. Transplant. 18, 669–678. 10.1111/ajt.14518 28960723

[B23] Han S. Ha S. Y. Park C. K. Joh J. W. Kwon C. H. Kwon G. Y. (2015). Microsteatosis may not interact with macrosteatosis in living donor liver transplantation. J. Hepatol. 62, 556–562. 10.1016/j.jhep.2014.10.027 25450710

[B24] Han S. Kim G. Lee S. K. Kwon C. H. Gwak M. Lee S.. (2014a). Comparison of the tolerance of hepatic ischemia/reperfusion injury in living donors: macrosteatosis versus microsteatosis. Liver Transplant. 20, 775–783. 10.1002/lt.23878 24687802

[B25] Han S. Ko J. S. Kwon G. Park C. Lee S. Kim J.. (2014b). Effect of pure microsteatosis on transplant outcomes after living donor liver transplantation: a matched case-control study. Liver Transplant. 20, 473–482. 10.1002/lt.23824 24425681

[B26] Higgins J. P. Thompson S. G. (2004). Controlling the risk of spurious findings from meta-regression. Stat. Med. 23, 1663–1682. 10.1002/sim.1752 15160401

[B27] Higgins J. P. Thompson S. G. Deeks J. J. Altman D. G. (2003). Measuring inconsistency in meta-analyses. BMJ 327, 557–560. 10.1136/bmj.327.7414.557 12958120 PMC192859

[B28] Homeyer A. Nasr P. Engel C. Kechagias S. Lundberg P. Ekstedt M.. (2017). Automated quantification of steatosis: agreement with stereological point counting. Diagn. Pathol. 12:80. 10.1186/s13000-017-0671-y 29132399 PMC5683532

[B29] Imber C. J. St. Peter S. D. Handa A. Friend P. J. (2002). Hepatic steatosis and its relationship to transplantation. Liver Transplant. 8, 415–423. 10.1053/jlts.2002.32275 12004340

[B30] Jackson D. White I. R. Thompson S. G. (2010). Extending DerSimonian and Laird's methodology to perform multivariate random effects meta-analyses. Stat. Med. 29, 1282–1297. 10.1002/sim.3602 19408255

[B31] Kim W. R. Biggins S. W. Kremers W. K. Wiesner R. H. Kamath P. S. Benson J. T.. (2008). Hyponatremia and mortality among patients on the liver-transplant waiting list. N. Eng. J. Med. 359, 1018–1026. 10.1056/NEJMoa0801209 18768945 PMC4374557

[B32] Koneru B. Dikdan G. (2002). Hepatic steatosis and liver transplantation current clinical and experimental perspectives. Transplantation 73, 325–330. 10.1097/00007890-200202150-00001 11884924

[B33] Kulik U. Lehner F. Klempnauer J. Borlak J. (2017). Primary non-function is frequently associated with fatty liver allografts and high mortality after re-transplantation. Liver Int. 37, 1219–1228. 10.1111/liv.13404 28267886

[B34] Li J. Liu B. Yan L. N. Zuo Y. X. Li B. Zeng Y. (2009). Reversal of graft steatosis after liver transplantation: prospective study. Transplant. Proc. 41, 3560–3563. 10.1016/j.transproceed.2009.06.222 19917344

[B35] Loinaz C. Gonzalez E. (2000). Marginal donors in liver transplantation. Hepatogastroenterology 47, 256–263. Available online at: https://www.ncbi.nlm.nih.gov/pubmed/10690618 10690618

[B36] Lozanovski V. J. Khajeh E. Fonouni H. Pfeiffenberger J. von Haken R. Brenner T.. (2018). The impact of major extended donor criteria on graft failure and patient mortality after liver transplantation. Langenbecks Arch. Surg. 403, 719–731. 10.1007/s00423-018-1704-z 30112639

[B37] Lucidi V. Gustot T. Moreno C. Donckier V. (2015). Liver transplantation in the context of organ shortage: toward extension and restriction of indications considering recent clinical data and ethical framework. Curr. Opin. Crit. Care 21, 163–170. 10.1097/MCC.0000000000000186 25692807

[B38] McCormack L. Dutkowski P. El-Badry A. M. Clavien P.-A. (2011). Liver transplantation using fatty livers: always feasible? J. Hepatol. 54, 1055–1062. 10.1016/j.jhep.2010.11.004 21145846

[B39] Moher D. Liberati A. Tetzlaff J. Altman D. G. (2009). Preferred reporting items for systematic reviews and meta-analyses: the PRISMA statement. Ann. Intern. Med. 151, 264–269. 10.7326/0003-4819-151-4-200908180-00135 19622511

[B40] Moosburner S. Gassner J. Nosser M. Pohl J. Wyrwal D. Claussen F.. (2018). Prevalence of steatosis hepatis in the eurotransplant region: impact on graft acceptance rates. HPB Surg. 2018:6094936. 10.1155/2018/6094936 30515073 PMC6236971

[B41] Neves D. B. Rusi M. B. Diaz L. G. G. Salvalaggio P. (2016). Primary graft dysfunction of the liver: definitions, diagnostic criteria and risk factors. Einstein 14, 567–572. 10.1590/s1679-45082016rw3585 27783749

[B42] Nikeghbalian S. Nejatollahi S. M. Salahi H. Bahador A. Sabet B. Jalaeian H.. (2007). Does donor's fatty liver change impact on early mortality and outcome of liver transplantation. Transplant. Proc. 39, 1181–1183. 10.1016/j.transproceed.2007.04.014 17524926

[B43] Nocito A. El-Badry A. M. Clavien P. A. (2006). When is steatosis too much for transplantation? J. Hepatol. 45, 494–499. 10.1016/j.jhep.2006.07.017 16919359

[B44] Noujaim H. M. de Ville de Goyet J. Montero E. F. Ribeiro C. M. Capellozzi V. L. Crescentini F.. (2009). Expanding postmortem donor pool using steatotic liver grafts: a new look. Transplantation 87, 919–925. 10.1097/TP.0b013e31819b3f76 19300197

[B45] Orsini N. Bellocco R. Greenland S. (2006). Generalized least squares for trend estimation of summarized dose-response data. Stata J. 6, 40–57. 10.1177/1536867X0600600103

[B46] Orsini N. Li R. Wolk A. Khudyakov P. Spiegelman D. (2011). Meta-analysis for linear and nonlinear dose-response relations: examples, an evaluation of approximations, and software. Am. J. Epidemiol. 175, 66–73. 10.1093/aje/kwr265 22135359 PMC3244608

[B47] Pais R. Barritt A. S. IV. Calmus Y. Scatton O. Runge T. Lebray P.. (2016). NAFLD and liver transplantation: current burden and expected challenges. J. Hepatol. 65, 1245–1257. 10.1016/j.jhep.2016.07.033 27486010 PMC5326676

[B48] Selzner N. Selzner M. Jochum W. Amann-Vesti B. Graf R. Clavien P. A. (2006). Mouse livers with macrosteatosis are more susceptible to normothermic ischemic injury than those with microsteatosis. J. Hepatol. 44, 694–701. 10.1016/j.jhep.2005.07.032 16229921

[B49] Spitzer A. L. Lao O. B. Dick A. A. Bakthavatsalam R. Halldorson J. B. Yeh M. M.. (2010). The biopsied donor liver: incorporating macrosteatosis into high-risk donor assessment. Liver Transplant. 16, 874–884. 10.1002/lt.22085 20583086

[B50] Tannapfel A. Denk H. Dienes H. P. Langner C. Schirmacher P. Trauner M.. (2011). Histopathological diagnosis of non-alcoholic and alcoholic fatty liver disease. Virchows Arch. 458, 511–523. 10.1007/s00428-011-1066-1 21442288

[B51] Urena M. A. Ruiz-Delgado F. C. Gonzalez E. M. Segurola C. L. Romero C. J. Garcia I. G.. (1998). Assessing risk of the use of livers with macro and microsteatosis in a liver transplant program. Transplant. Proc. 30, 3288–3291. 10.1016/S0041-1345(98)01033-1 9838454

[B52] Verran D. Kusyk T. Painter D. Fisher J. Koorey D. Strasser S.. (2003). Clinical experience gained from the use of 120 steatotic donor livers for orthotopic liver transplantation. Liver Transplantat. 9, 500–505. 10.1053/jlts.2003.50099 12740794

[B53] Vinaixa C. Selzner N. Berenguer M. (2018). Fat and liver transplantation: clinical implications. Transplant. Int. 31, 828–837. 10.1111/tri.13288 29883530

[B54] Vodkin I. Kuo A. (2017). Extended criteria donors in liver transplantation. Clin. Liver Dis. 21, 289–301. 10.1016/j.cld.2016.12.004 28364814

[B55] Wells G. Shea B. O'connell D. Peterson J. Welch V. Losos M. (2016). The Newcastle-Ottawa Scale (NOS) for Assessing the Quality of Nonrandomised Studies in Meta-Analyses. Ottawa, ON: Ottawa Hospital Research Institute.

[B56] Westerkamp A. C. de Boer M. T. van den Berg A. P. Gouw A. S. Porte R. J. (2015). Similar outcome after transplantation of moderate macrovesicular steatotic and nonsteatotic livers when the cold ischemia time is kept very short. Transplant. Int. 28, 319–329. 10.1111/tri.12504 25545740

[B57] Wong T. C. Fung J. Y. Chok K. S. Cheung T. T. Chan A. C. Sharr W. W.. (2016). Excellent outcomes of liver transplantation using severely steatotic grafts from brain-dead donors. Liver Transplant. 22, 226–236. 10.1002/lt.24335 26359934

[B58] Younossi Z. Anstee Q. M. Marietti M. Hardy T. Henry L. Eslam M.. (2018). Global burden of NAFLD and NASH: trends, predictions, risk factors and prevention. Nat. Rev. Gastroenterol. Hepatol. 15, 11–20. 10.1038/nrgastro.2017.109 28930295

