# Supplementary material for: Systematic Evaluation of the Safety Threshold for Allograft Macrovesicular Steatosis in Cadaveric Liver Transplantation
Source: Front Physiol. 2019 Apr 25;10:429. doi: 10.3389/fphys.2019.00429 (PMC6494939; doi:10.3389/fphys.2019.00429)
Supplement: Supplementary file 1 [file Data_Sheet_1.docx]

**Supplementary tables**

Table S1 Literature search strategy across different databases

| **ISI-web of science** | Indexes=SCI-EXPANDED, SSCI, A&HCI, CPCI-S, CPCI-SSH, ESCI, CCR-EXPANDED, IC Timespan=1975-2018 |  |
| --- | --- | --- |
| #1 | (TS=(steatosis)) AND DOCUMENT TYPES: (Article) | 17033 |
| #2 | (TS=(macrosteatosis)) AND DOCUMENT TYPES: (Article) | 113 |
| #3 | (TS=(fatty liver)) AND DOCUMENT TYPES: (Article) | 48236 |
| #4 | \| (TS=(non-alcoholic fatty liver disease)) AND DOCUMENT TYPES: (Article) \| \| --- \| | 5230 |
| #5 | (TS=(NAFLD)) AND DOCUMENT TYPES: (Article) | 7298 |
| #6 | (TS=(NASH)) AND DOCUMENT TYPES: (Article) | 18681 |
| #7 | #1 OR #2 OR #3 OR #4 OR #5 OR #6 | 69869 |
| #8 | (TS=(liver transplantation)) AND DOCUMENT TYPES: (Article) | 61890 |
| #9 | (TS=(mortality)) AND DOCUMENT TYPES: (Article) | 618360 |
| #10 | (TS=(survival)) AND DOCUMENT TYPES: (Article) | 810694 |
| #11 | #9 OR #10 | 1298451 |
| #12 | #7 AND #8 AND #11 | 675 |
|  |  |  |
| **Embase** |  |  |
| #1 | steatosis {Including Related Terms} (limit to full text) | 1612 |
| #2 | macrosteatosis {Including Related Terms} (limit to full text) | 139 |
| #3 | fatty liver {Including Related Terms} (limit to full text) | 2660 |
| #4 | non-alcoholic fatty liver disease {Including Related Terms} (limit to full text) | 3206 |
| #5 | NAFLD {Including Related Terms} (limit to full text) | 579 |
| #6 | NASH {Including Related Terms} (limit to full text) | 298 |
| #7 | (“steatosis” or “macrosteatosis” or “fatty liver” or “NAFLD” or “NASH” or “non-alcoholic fatty liver disease”) {Including Related Terms} (limit to full text) | 2542 |
| #8 | liver transplantation {Including Related Terms} (limit to full text) | 13075 |
| #9 | mortality {Including Related Terms} (limit to full text) | 32744 |
| #10 | survival {Including Related Terms} (limit to full text) | 15131 |
| #11 | (“mortality” or “survival”) {Including Related Terms} (limit to full text) | 6487 |
| #12 | #7 AND #8 AND #11 | 3323 |
|  |  |  |
| **PubMed** |  |  |
| #1 | Search steatosis Filters: Full text | 58541 |
| #2 | Search macrosteatosis Filters: Full text | 117 |
| #3 | Search fatty liver Filters: Full text | 54016 |
| #4 | Search non-alcoholic fatty liver disease Filters: Full text | 11892 |
| #5 | Search NAFLD Filters: Full text | 13753 |
| #6 | Search NASH Filters: Full text | 12132 |
| #7 | Search (((((steatosis) OR macrosteatosis) OR fatty liver) OR non-alcoholic fatty liver disease) OR NAFLD) OR NASH Filters: Full text | 66571 |
| #8 | Search liver transplantation Filters: Full text | 77935 |
| #9 | Search mortality Filters: Full text | 843152 |
| #10 | Search survival Filters: Full text | 1385692 |
| #11 | Search (mortality) OR survival Filters: Full text | 1439421 |
| #12 | Search ((((((steatosis) OR macrosteatosis) OR fatty liver) OR non-alcoholic fatty liver disease) OR NAFLD) OR NASH) AND (liver transplantation) AND ((mortality) OR survival) Filters: Full text | 892 |
|  |  |  |

Table S2 Newcastle-Ottawa scale (NOS) scale for Quality Assessment on Included Studies

|  | Deroose, Netherland, 2011 | Graaf, Australia, 2011 | Doyle, USA, 2010 | Noujaim, Brazil, 2009 | Li,  China, 2009 | Burra, Italy, 2009 | Nikeghbalian, Iran,  2007 | Briceno,  Spain,  2005 | Verran, Australia, 2003 |
| --- | --- | --- | --- | --- | --- | --- | --- | --- | --- |
| **Selection** |  |  |  |  |  |  |  |  |  |
| Representativeness of the cohort using MaS allografts | 1 | 1 | 1 | 1 | 1 | 1 | 1 | 1 | 1 |
| Selection of the cohort using non-MaS allografts | 1 | 1 | 1 | 1 | 1 | 1 | 1 | 1 | 1 |
| Ascertainment of MaS allografts | 1 | 1 | 1 | 1 | 1 | 1 | 1 | 1 | 1 |
| Demonstration that inferior outcomes were excluded or distinguished at start of study | 1 | 1 | 1 | 1 | 1 | 1 | 1 | 1 | 1 |
| **Comparison** |  |  |  |  |  |  |  |  |  |
| Controls for recipient MELD score | 0 | 1 | 1 | 1 | 1 | 1 | 0 | 1 | 0 |
| Controls for cold ischemic time | 0 | 1 | 1 | 1 | 1 | 1 | 0 | 1 | 0 |
| **Outcome** |  |  |  |  |  |  |  |  |  |
| Assessment of posttransplant outcomes | 1 | 1 | 1 | 1 | 1 | 1 | 1 | 1 | 1 |
| Long enough follow-up for evaluation of posttransplant outcomes | 1 | 1 | 1 | 0 | 0 | 1 | 0 | 0 | 0 |
| Adequacy of follow up of cohorts | 0 | 0 | 0 | 0 | 0 | 0 | 0 | 0 | 0 |
| Total (9 as maximum) | 6 | 8 | 8 | 7 | 7 | 8 | 5 | 7 | 5 |

Abbreviations: MaS, macrovesicular steatosis; MELD, model for end-stage liver disease.

Table S3 Major indicators observed in enrolled studies

| Author, year | Patients’ Survival | | | | |  | Organ Failure | | | | |  | Complications | |  |  |  |
| --- | --- | --- | --- | --- | --- | --- | --- | --- | --- | --- | --- | --- | --- | --- | --- | --- | --- |
|  | 90 day | 1 year | 2 year | 3 year | 5 year |  | 90 day | 1 year | 2 year | 3 year | 5 year |  | EAD | PNF |  | Liver enzyme | Length of Hospitalization |
| Graaf, Australia, 2012 | yes | na | na | na | na |  | na | yes | yes | yes | yes |  | yes | yes |  | na | na |
| Deroose, Netherland, 2011 | yes | yes | yes | yes | yes |  | yes | yes | yes | yes | yes |  | yes | yes |  | ALT/AST | In ward/ICU stay |
| Doyle, USA, 2010 | yes | yes | yes | yes | yes |  | yes | yes | yes | yes | yes |  | na | yes |  | ALT/AST | In ward/ICU stay |
| Noujaim, Brazil, 2009 | yes | yes | na | na | na |  | yes | yes | yes | na | na |  | na | na |  | AST | na |
| Li, China, 2009 | na | yes | yes | na | na |  | na | yes | yes | na | na |  | yes | yes |  | ALT/AST | In ward/ICU stay |
| Burra, Italy, 2009 | yes | yes | yes | yes | na |  | na | na | na | na | na |  | na | yes |  | na | na |
| Nikeghbalian, Iran, 2007 | yes | yes | na | na | na |  | na | na | na | na | na |  | na | na |  | na | In ward/ICU stay |
| Briceno, Spain, 2005 | yes | na | na | na | na |  | yes | na | na | na | na |  | na | yes |  | na | na |
| Verran, Australia, 2003 | na | na | na | na | na |  | yes | yes | yes | yes | yes |  | yes | yes |  | na | na |

Abbreviation: ALT, alanine aminotransferase; AST, aspart aminotransferase; EAD, early allograft dysfunction; ICU, Intensive Care Unit; na, not available; PNF, primary non-function.

Table S4 Cause of patient death and organ failure reported in enrolled studies

| Cause of incidence | Patient Feature | Indicator/Case Number (%^~~a~~^) | Time after liver transplantation (year) |
| --- | --- | --- | --- |
| Graaf et al, 2012 | Non-steatostic graft | Patient Death | 3 months |
| Sepsis |  | 1(25) |  |
| Cardiac arrest |  | 1(25) |  |
| MOF |  | 2(50) |  |
|  | Steatostic graft |  |  |
| Sepsis |  | 2(40) |  |
| MOF |  | 1(20) |  |
| Cardiac arrest |  | 1(20) |  |
| Others |  | 1(20) |  |
| Deroose et al, 2011 | Whole group | Graft loss | 6 months |
| Billiary duct  ischaemia |  | 6(43) |  |
| Hepatic artery  thrombosis |  | 5(36) |  |
| Smallfor-size graft |  | 2(14) |  |
| Chronic rejection |  | 1(7) |  |
| Noujaim et al, 2009 | Non-steatotic graft | Patient Death | 3 years |
| Cardiac disease |  | 2(33) |  |
| Malignancy |  | 2(33) |  |
| Sepsis |  | 2(33) |  |
|  | Steatotic graft | Patient Death | 3 years |
| Spesis |  | 3(50) |  |
| HCV recurrence |  | 2(33) |  |
| Bleeding |  | 1(17) |  |
| Verran et al, 2003 | Steatotic graft | Graft loss | 1 year |
| Rejection |  | 17(23) |  |
| Patient death |  | 35(48) |  |
| Primary disease recurrence |  | 7(10) |  |
| Hepatic artery thrombosis |  | 10(14) |  |
| Others |  | 4(5) |  |
|  | Non-steatotic graft | Graft loss |  |
| Rejection |  | 5(14) |  |
| Patient death |  | 21(60) |  |
| Primary disease recurrence |  | 5(14) |  |
| Hepatic artery thrombosis |  | 2(6) |  |
| Others |  | 2(6) |  |

^a^ represented the percentage of case compared to the total incidence in the same period.

Abbreviation: HCV, hwpatitis C virus; MOF, multiple organ failure; PNF, primary nonfunction.

Table S5 Categorical comparison on continuous covariates across subgroups classified by macrosteatosis degree

| Covariate | Comparison (MaS degree) | Number of studies | Number of patients | SMD | I^2^(%) | p-value (Heterogeneity chi-squared) | p-value (Egger’s test) |
| --- | --- | --- | --- | --- | --- | --- | --- |
| Recipient age | High vs. low | 5 | 694 | -0.12(-0.37/0.14) | 10.1 | 0.35 | 0.08 |
|  | Middle vs. low |  |  | 0.07(-0.10/0.25) | 0.3 | 0.40 | 0.67 |
| Donor age | High vs. low | 5 | 694 | -0.15(-0.41/0.11) | 77.9 | <0.01 | 0.11 |
|  | Middle vs. low |  |  | 0.49(0.31-0.66) | 59.7 | 0.04 | 0.50 |
| Recipient BMI | High vs. low | 2 | 380 | 0.25(-0.11/0.60) | 0 | 0.96 | NA |
|  | Middle vs. low |  |  | 0.13(-0.11/0.38) | 78.2 | 0.03 | NA |
| Donor BMI | High vs. low | 4 | 578 | 0.40(0.11-0.68) | 73.1 | 0.01 | 0.41 |
|  | Middle vs. low |  |  | 0.31(0.12-0.51) | 72.3 | 0.01 | 0.72 |
| MELD score | High vs. low | 6 | 1194 | -0.36(-0.54/-0.18) | 73.1 | <0.01 | 0.15 |
|  | Middle vs. low |  |  | 0(-0.13/0.13) | 0 | 0.92 | 0.14 |
| Cold ischemic time | High vs. low | 6 | 1194 | -0.35(-0.53/-0.17) | 63.8 | 0.02 | 0.02 |
|  | Middle vs. low |  |  | 0.04(-0.09/0.17) | 38.9 | 0.15 | 0.84 |

Data in extremely high MaS group (MaS>60%) was combined into prior group for comparison.

NA in Egger’s test was for less than three studies combined for comparison.

Abbreviation: BMI, body mass index; MaS, macrovesicular steatosis; MELD, model for end-stage liver disease; SMD, standardized mean difference

Table S6 Definition of early allograft dysfunction and primary nonfunction in enrolled studies

| Study | EAD | PNF |
| --- | --- | --- |
| Deroose et al, 2011 | Met one or more following criteria in first week after liver transplantation: | na |
|  | 1. TB>10 mg/dl |  |
|  | 2. INR≥1.6 |  |
|  | 3. ALT or AST> 2000 IU/ml |  |
|  |  |  |
| Graaf et al, 2011 | Met one criteria for two consecutive measurements in first 72 hours after OLT, with elevation lasting for 48 hours | Initial poor function of the allograft during the first week after OLT, culminating in either death of the recipient or retransplantation. |
|  | 1. ALT >1500 U/mL or |  |
|  | 2. AST >1500 U/mL |  |
|  |  |  |
| Noujaim et al, 2009 | Met one or more following criteria in first week after liver transplantation: | Acute syndrome with: |
|  | 1. AST> 2000 IU/ml | 1. Clinical encephalopathy, |
|  | 2. PT<50% of normal value | 2. Hemodynamic instability with need for inotropic support, hypoglycemia and glucose replacement, |
|  |  | 3. AST>2000U/L, |
|  |  | 4. Metabolic acidosis, renal failure, and urgent need of retransplantation |
|  |  |  |
| Burra et al,2009 | NA | Numerical score of 0 to 2 was awarded to each parameter, 1. AST in 18 hours after transplantation [(0) <1500 IU/L, (1) 1500-2200 IU/L, and (2) >2200 IU/L],  2. coagulation factor V in 24 hours [(0) <35%, (1) 25%-35%, and (2) >25%],  3. PT in 36 hours [(0) >25%, (1) 15%-25%, and (2) <15%].  Primary nonfunction was scored as 5 or 6. |
|  |  |  |
| Briceno et al, 2005 | Met one characteristic during the first postoperative week | Nonrecoverable hepatocellular function necessitating emergency retransplantation within the first postoperative 72 hours. |
|  | 1. ALT > 1000 U/l |  |
|  | 2. AST > 1500 U/l |  |
|  |  |  |
| Verran et al, 2003 | Met one criteria for two consecutive measurements in the first 72 hours after liver transplantation | Defined as poor function of the allograft culminating in recipient death or retransplantation. |
|  | 1. ALT >1500 U/mL or |  |
|  | 2. AST >1500 U/mL |  |

Abbreviation: ALT, alanine aminotransferase; AST, aspart aminotransferase; EAD, early allograft dysfunction; INR, international normalized ratio;

na, not available; OLT, orthotopic liver transplantation; PNF, primary nonfunction; PT, prothrombin time; TB, total bilirubin.

Table S7 Meta-regression analysis on impacts of potential confounders on MaS-outcomes association

| Items | Patient mortality | | |  | Allograft failure | | |  | Post-transplant outcome |
| --- | --- | --- | --- | --- | --- | --- | --- | --- | --- |
|  | 90-day | 1-year | 2-year |  | 90-day | 1-year | 2-year |  | PNF |
| Sample size |  |  |  |  |  |  |  |  |  |
| Number of studies | 7 | 6 | 4 |  | 5 | 6 | 5 |  | 7 |
| Number of patients | 1463 | 892 | 661 |  | 1475 | 1186 | 1129 |  | 1745 |
| Coefficient(b) | 1.00 | 1.00 | 1.00 |  | 1.00 | 1.00 | 1.00 |  | 1.00 |
| P-value | 0.56 | 0.82 | 0.39 |  | 0.91 | 0.95 | 0.89 |  | 0.93 |
|  |  |  |  |  |  |  |  |  |  |
| Recipient age |  |  |  |  |  |  |  |  |  |
| Number of studies | 4 | 4 | 3 |  | na | 4 | 3 |  | 4 |
| Number of patients | 624 | 553 | 496 |  |  | 578 | 521 |  | 637 |
| Coefficient(b) | 1.65 | 0.69 | 0.30 |  |  | 3.35 | 0.83 |  | 1.07 |
| P-value | 0.77 | 0.80 | 0.50 |  |  | 0.60 | 0.94 |  | 0.99 |
|  |  |  |  |  |  |  |  |  |  |
| Donor age |  |  |  |  |  |  |  |  |  |
| Number of studies | 4 | 4 | 3 |  | na | 4 | 3 |  | 4 |
| Number of patients | 624 | 624 | 496 |  |  | 578 | 521 |  | 637 |
| Coefficient(b) | 1.15 | 0.81 | 0.55 |  |  | 1.68 | 0.87 |  | 1.05 |
| P-value | 0.87 | 0.77 | 0.50 |  |  | 0.65 | 0.90 |  | 0.98 |
|  |  |  |  |  |  |  |  |  |  |
| Donor BMI |  |  |  |  |  |  |  |  |  |
| Number of studies | 3 | 3 | na |  | na | 4 | 3 |  | 3 |
| Number of patients | 625 | 508 |  |  |  | 578 | 521 |  | 521 |
| Coefficient(b) | 0.45 | 1.45 |  |  |  | 1.12 | 0.90 |  | 8.98 |
| P-value | 0.71 | 0.84 |  |  |  | 0.90 | 0.91 |  | 0.46 |
|  |  |  |  |  |  |  |  |  |  |
| MELD score |  |  |  |  |  |  |  |  |  |
| Number of studies | 5 | 4 | 3 |  | 3 | 4 | 3 |  | 5 |
| Number of patients | 1124 | 553 | 496 |  | 867 | 578 | 521 |  | 1137 |
| Coefficient(b) | 2.25 | 0.14 | 0.19 |  | 0.70 | 0.67 | 0.79 |  | 0.05 |
| P-value | 0.35 | 0.52 | 0.73 |  | 0.93 | 0.76 | 0.87 |  | 0.17 |
|  |  |  |  |  |  |  |  |  |  |
| Cold ischemic time |  |  |  |  |  |  |  |  |  |
| Number of studies | 5 | 4 | 3 |  | 3 | 4 | 3 |  | 5 |
| Number of patients | 1124 | 553 | 496 |  | 867 | 578 | 521 |  | 1137 |
| Coefficient(b) | 2.20 | 0.07 | 0.23 |  | 0.93 | 0.77 | 2.78 |  | 0.40 |
| P-value | 0.40 | 0.51 | 0.74 |  | 0.99 | 0.91 | 0.72 |  | 0.71 |

“na” represented non-available results for less than three data points collected in meta-regression analysis.

Abbreviation: BMI, body mass index; MELD, model for end-stage liver disease; PNF, primary nonfunction.
