# Supplementary material for: Systematic Evaluation of the Safety Threshold for Allograft Macrovesicular Steatosis in Cadaveric Liver Transplantation
Source: Front Physiol. 2019 Apr 25;10:429. doi: 10.3389/fphys.2019.00429 (PMC6494939; doi:10.3389/fphys.2019.00429)
Supplement: Supplementary file 2 [file Data_Sheet_2.docx]

**Supplementary Figures**

**Figure Legend**

Figure S1 Impact of donor MaS severity on liver function or inhospitalization in each individual study

A. Impact of MaS severity on peak post-operational ALT level for patients after liver transplantation;

B. Impact of MaS severity on peak post-operational AST level for patients after liver transplantation;

C. Impact of MaS severity on peak post-operational time of ward stay for patients after liver transplantation;

D. Impact of MaS severity on peak post-operational time of ICU stay for patients after liver transplantation;

* represented significant change compared between tagged and control group (P<0.05).

ALT, alanine aminotransferase; AST, aspart aminotransferase; ICU, Intensive Care Unit; MaS,macrovesicular steatosis.

Figure S2 Sensitivity analysis on pooled risk of patient mortality in groups using allografts with high and low MaS severity.

A represented sensitivity analysis on pooled risk of 90-day patient mortality

(high vs. low);

B represented sensitivity analysis on pooled risk of 1-year patient mortality

(high vs. low);

C represented sensitivity analysis on pooled risk of 2-year patient mortality

(high vs. low);

D represented sensitivity analysis on pooled risk of 3-year patient mortality

(high vs. low).

Abbreviation: MaS,macrovesicular steatosis.

Figure S3 Sensitivity analysis on pooled risk of graft failure in groups using allografts with high and low MaS severity.

A represented sensitivity analysis on pooled risk of 90-day graft failure

(high vs. low);

B represented sensitivity analysis on pooled risk of 1-year graft failure

(high vs. low);

C represented sensitivity analysis on pooled risk of 2-year graft failure

(high vs. low);

D represented sensitivity analysis on pooled risk of 3-year graft failure

(high vs. low).

Abbreviation: MaS,macrovesicular steatosis.

Figure S4 Sensitivity analysis on pooled risk of post-transplant complications in groups using allografts with high and low MaS severity.

A represented sensitivity analysis on pooled risk of PNF occurrence (high vs. low);

B represented sensitivity analysis on pooled risk of EAD occurrence (high vs. low).

Abbreviation: EAD, early allograft dysfunction; MaS,macrovesicular steatosis; PNF, primary nonfunction.

Figure S1


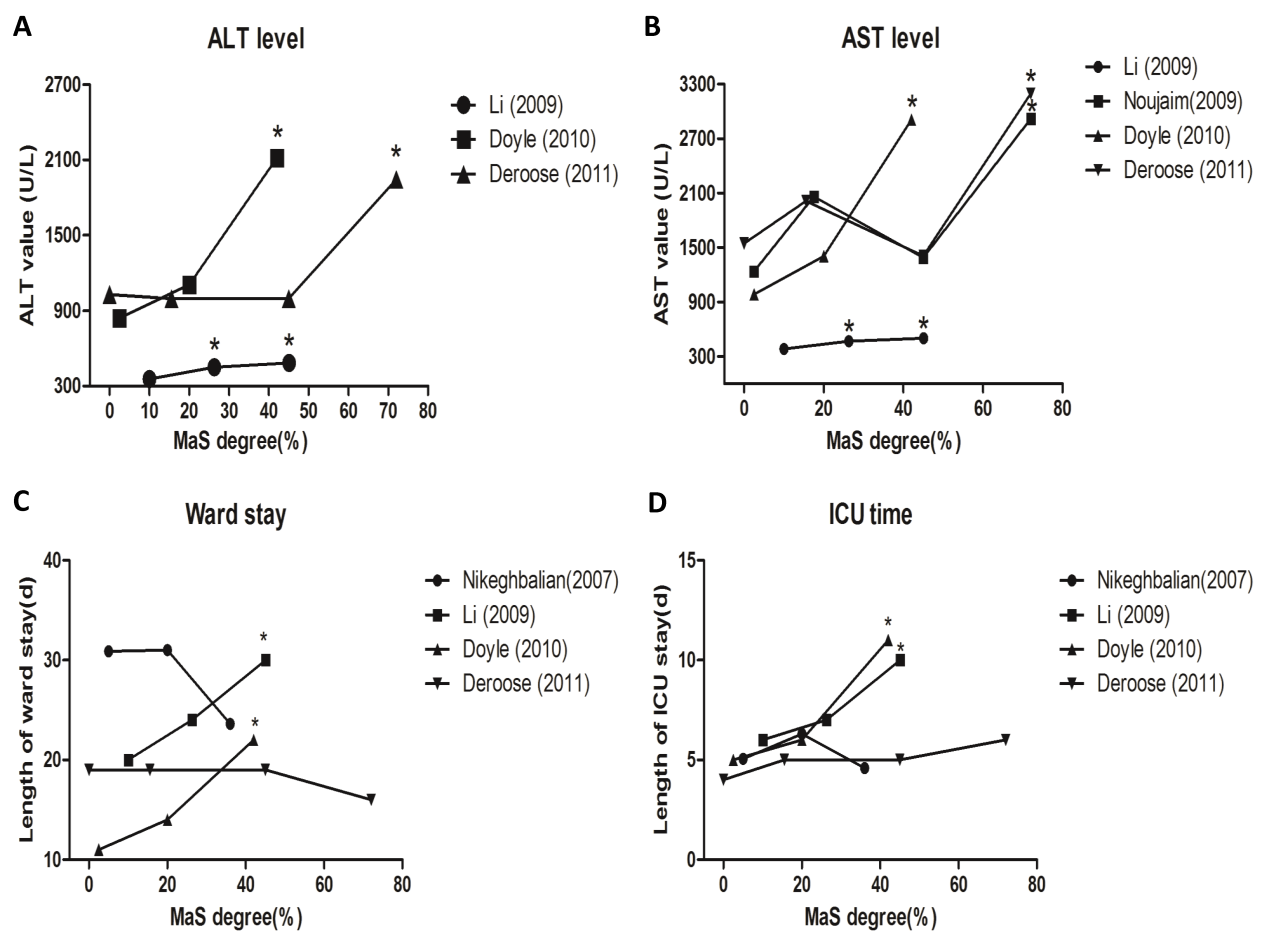


Figure S2



Figure S3





Figure S4
