# Supplementary material for: Systematic Evaluation of the Safety Threshold for Allograft Macrovesicular Steatosis in Cadaveric Liver Transplantation
Source: Front Physiol. 2019 Apr 25;10:429. doi: 10.3389/fphys.2019.00429 (PMC6494939; doi:10.3389/fphys.2019.00429)
Supplement: Supplementary file 3 [file Data_Sheet_3.docx]

**NOS checklist on quality assessment of enrolled studies related to risk of donor MaS on posttransplant outcomes based on modified NOS scale**

**Selection**

1) Representativeness of the cohort using MaS allografts

a) truly representative of general subjects in the community **🟑**

b) somewhat representative of general subjects in the community **🟑**

c) selected group of subjects

d) no description on the derivation of the cohort

2) Selection of the cohort using non-MaS allografts

a) drawn from the same community as exposed cohort**🟑**

b) drawn from a different source

c) no description of the derivation of the non exposed cohort

3) Ascertainment of MaS allografts

a) secure record (pathological examination) **🟑**

b) written self report

c) no description

4) Demonstration that inferior outcomes were excluded or distinguished at start of study

a) yes **🟑**

b) no

**Comparability**

1) Comparability of cohorts on the basis of the study design

a) study controls for recipient MELD score**🟑**

b) study controls for cold ischemic time **🟑**

**Outcome**

1) Assessment of posttransplant outcomes

a) definite outcome criteria **🟑**

b) record linkage **🟑**

c) self report

d) no description

2) Was follow-up long enough for evaluation of posttransplant outcomes

a) yes (if follow up period>3 years) **🟑**

b) no

3) Adequacy of follow up of cohorts

a) complete follow up for all subjects **🟑**

b) cohorts with follow up rate≥80% **🟑**

c) cohorts with follow up rate < 80%

d) no statement
